# Lipoprotein signatures of cholesteryl ester transfer protein and HMG-CoA reductase inhibition

**DOI:** 10.1371/journal.pbio.3000572

**Published:** 2019-12-20

**Authors:** Johannes Kettunen, Michael V. Holmes, Elias Allara, Olga Anufrieva, Pauli Ohukainen, Clare Oliver-Williams, Qin Wang, Therese Tillin, Alun D. Hughes, Mika Kähönen, Terho Lehtimäki, Jorma Viikari, Olli T. Raitakari, Veikko Salomaa, Marjo-Riitta Järvelin, Markus Perola, George Davey Smith, Nish Chaturvedi, John Danesh, Emanuele Di Angelantonio, Adam S. Butterworth, Mika Ala-Korpela

**Affiliations:** 1 Computational Medicine, Faculty of Medicine, University of Oulu and Biocenter Oulu, Oulu, Finland; 2 National Institute for Health and Welfare, Helsinki, Finland; 3 Medical Research Council Population Health Research Unit, University of Oxford, Oxford, United Kingdom; 4 Clinical Trial Service Unit and Epidemiological Studies Unit, Nuffield Department of Population Health, University of Oxford, Oxford, United Kingdom; 5 National Institute for Health Research, Oxford Biomedical Research Centre, Oxford University Hospital, Oxford, United Kingdom; 6 Medical Research Council Integrative Epidemiology Unit at the University of Bristol, Bristol, United Kingdom; 7 British Heart Foundation Cardiovascular Epidemiology Unit, Department of Public Health and Primary Care, University of Cambridge, Cambridge, United Kingdom; 8 National Institute for Health Research Blood and Transplant Research Unit in Donor Health and Genomics, University of Cambridge, Cambridge, United Kingdom; 9 Homerton College, University of Cambridge, Cambridge, United Kingdom; 10 Institute of Cardiovascular Science, University College London, London, United Kingdom; 11 Department of Clinical Physiology, University of Tampere and Tampere University Hospital, Tampere, Finland; 12 Department of Clinical Chemistry, Fimlab Laboratories, Finnish Cardiovascular Research Center Tampere, Faculty of Medicine and Health Technologies, University of Tampere, Tampere, Finland; 13 Department of Medicine, University of Turku, Turku, Finland; 14 Division of Medicine, Turku University Hospital, Turku, Finland; 15 Research Centre of Applied and Preventive Cardiovascular Medicine, University of Turku, Turku, Finland; 16 Department of Clinical Physiology and Nuclear Medicine, Turku University Hospital, Turku, Finland; 17 Center for Life Course Health Research, Faculty of Medicine, University of Oulu, Oulu, Finland; 18 Biocenter Oulu, University of Oulu, Oulu, Finland; 19 Unit of Primary Health Care, Oulu University Hospital, OYS, Oulu, Finland; 20 Department of Epidemiology and Biostatistics, MRC-PHE Centre for Environment and Health, School of Public Health, Imperial College London, London, United Kingdom; 21 Department of Life Sciences, College of Health and Life Sciences, Brunel University London, United Kingdom; 22 Diabetes and Obesity Research Program, University of Helsinki, Helsinki, Finland; 23 Estonian Genome Center, University of Tartu, Tartu, Estonia; 24 Population Health Science, Bristol Medical School, University of Bristol, Bristol, United Kingdom; 25 Wellcome Trust Sanger Institute, Hinxton, United Kingdom; 26 British Heart Foundation Cambridge Centre of Excellence, Department of Medicine, University of Cambridge, Cambridge, United Kingdom; 27 NMR Metabolomics Laboratory, School of Pharmacy, University of Eastern Finland, Kuopio, Finland; 28 Systems Epidemiology, Baker Heart and Diabetes Institute, Melbourne, Victoria, Australia; 29 Department of Epidemiology and Preventive Medicine, School of Public Health and Preventive Medicine, Faculty of Medicine, Nursing and Health Sciences, The Alfred Hospital, Monash University, Melbourne, Victoria, Australia; Newcastle University, UNITED KINGDOM

## Abstract

Cholesteryl ester transfer protein (CETP) inhibition reduces vascular event risk, but confusion surrounds its effects on low-density lipoprotein (LDL) cholesterol. Here, we clarify associations of genetic inhibition of CETP on detailed lipoprotein measures and compare those to genetic inhibition of 3-hydroxy-3-methylglutaryl-coenzyme A reductase (HMGCR). We used an allele associated with lower *CETP* expression (rs247617) to mimic CETP inhibition and an allele associated with lower *HMGCR* expression (rs12916) to mimic the well-known effects of statins for comparison. The study consists of 65,427 participants of European ancestries with detailed lipoprotein subclass profiling from nuclear magnetic resonance spectroscopy. Genetic associations were scaled to 10% reduction in relative risk of coronary heart disease (CHD). We also examined observational associations of the lipoprotein subclass measures with risk of incident CHD in 3 population-based cohorts totalling 616 incident cases and 13,564 controls during 8-year follow-up. Genetic inhibition of CETP and HMGCR resulted in near-identical associations with LDL cholesterol concentration estimated by the Friedewald equation. Inhibition of HMGCR had relatively consistent associations on lower cholesterol concentrations across all apolipoprotein B-containing lipoproteins. In contrast, the associations of the inhibition of CETP were stronger on lower remnant and very-low-density lipoprotein (VLDL) cholesterol, but there were no associations on cholesterol concentrations in LDL defined by particle size (diameter 18–26 nm) (−0.02 SD LDL defined by particle size; 95% CI: −0.10 to 0.05 for *CETP* versus −0.24 SD, 95% CI −0.30 to −0.18 for *HMGCR*). Inhibition of CETP was strongly associated with lower proportion of triglycerides in all high-density lipoprotein (HDL) particles. In observational analyses, a higher triglyceride composition within HDL subclasses was associated with higher risk of CHD, independently of total cholesterol and triglycerides (strongest hazard ratio per 1 SD higher triglyceride composition in very large HDL 1.35; 95% CI: 1.18–1.54). In conclusion, CETP inhibition does not appear to affect size-specific LDL cholesterol but is likely to lower CHD risk by lowering concentrations of other atherogenic, apolipoprotein B-containing lipoproteins (such as remnant and VLDLs). Inhibition of CETP also lowers triglyceride composition in HDL particles, a phenomenon reflecting combined effects of circulating HDL, triglycerides, and apolipoprotein B-containing particles and is associated with a lower CHD risk in observational analyses. Our results reveal that conventional composite lipid assays may mask heterogeneous effects of emerging lipid-altering therapies.

## Abstract 2

Inhibition of cholesteryl ester transfer protein does not affect size-specific low-density lipoprotein cholesterol, but may lower coronary heart disease risk by lowering cholesterol concentrations in other apolipoprotein-B containing atherogenic lipoproteins, and by lowering triglyceride content of high-density lipoprotein particles.

## Introduction

Definitive evidence on the causal role of low-density lipoproteins (LDLs) in cardiovascular disease comes from trials of LDL cholesterol lowering compounds [1], which have shown beneficial effects on risk of coronary heart disease (CHD) and stroke. Consistent effects have been seen for drugs acting on related pathways, such as 3-hydroxy-3-methylglutaryl-coenzyme A reductase (HMGCR) inhibitors, i.e., statins, and proprotein convertase subtilisin-kexin type 9 (PCSK9) inhibitors [2], both of which up-regulate hepatic LDL receptor expression, and for drugs acting on other pathways, such as ezetimibe [3], which inhibits intestinal absorption of cholesterol [4].

However, trials of drugs primarily designed to alter concentrations of lipids other than LDL cholesterol have had mixed results [5,6]. One such example is the class of drugs designed to inhibit cholesteryl ester transfer protein (CETP), a lipid transport protein responsible for the exchange of triglycerides and cholesteryl esters between apolipoprotein B-containing particles and high-density lipoprotein (HDL) particles. CETP inhibitors were developed initially on the basis of their HDL cholesterol raising effects. Although accumulating genetic evidence suggests that HDL cholesterol concentration is unlikely to be causally related to CHD [7–9], there were 2 strong reasons to believe that CETP inhibition may still reduce vascular risk: (i) genetic studies of *CETP* variants have shown associations with CHD [10,11] and (ii) some CETP inhibitors not only increase HDL cholesterol but also appear to lower LDL cholesterol as measured by conventional assays [12,13].

The findings from the phase III REVEAL study, the largest CETP trial to date, showed that treatment with the CETP inhibitor anacetrapib led to a reduction in risk of coronary events that was proportional to the reduction in non-HDL cholesterol [14]. Interestingly, anacetrapib appeared to have discrepant effects based on the assay used to quantify LDL cholesterol (using beta-quant, direct or Friedewald estimation) [13]. This discrepant effect was also identified in a genetic study that approximated a factorial clinical trial of CETP inhibition and statin therapy [15]. Thus, although both CETP inhibitors and statins lower Friedewald-estimated LDL cholesterol, which also includes cholesterol carried by other lipoprotein particles, it is possible that the drugs have differential effects on the concentration and composition of lipids in different apolipoprotein B–containing lipoproteins.

In this study, we used the established approach of exploiting genetic variants near the protein-coding genes of drug targets to investigate detailed lipid and lipoprotein subclass signatures of CETP inhibition in large population-based studies. We compared the association of an allele associated with lower *CETP* expression (to mimic CETP inhibition) with *HMGCR* expression associated variant (to proxy statin treatment) to gauge into how these 2 therapies alter the lipoprotein milieu. The genetic effects of *HMGCR* inhibition were analysed to provide a valuable control because these effects are already well understood [16]. We also examined the role of lipoprotein composition in CHD for the first time. We present findings that the triglyceride composition, in contrast to circulating concentrations, of HDL particles is associated with CHD.

## Results

Data from 62,400 individuals with extensive lipoprotein subclass profiling and genotypes were available. We combined data from 5 adult cohorts (mean age range from 31–52 years) and one cohort of adolescents (mean age 16 years) for the genetic analyses in which 51% of participants of all 6 studies were female. Study-specific and pooled estimates from meta-analyses of genetic and observational analyses for all 191 traits are presented in Supporting S1–S15 Figs.

**Fig 1:**
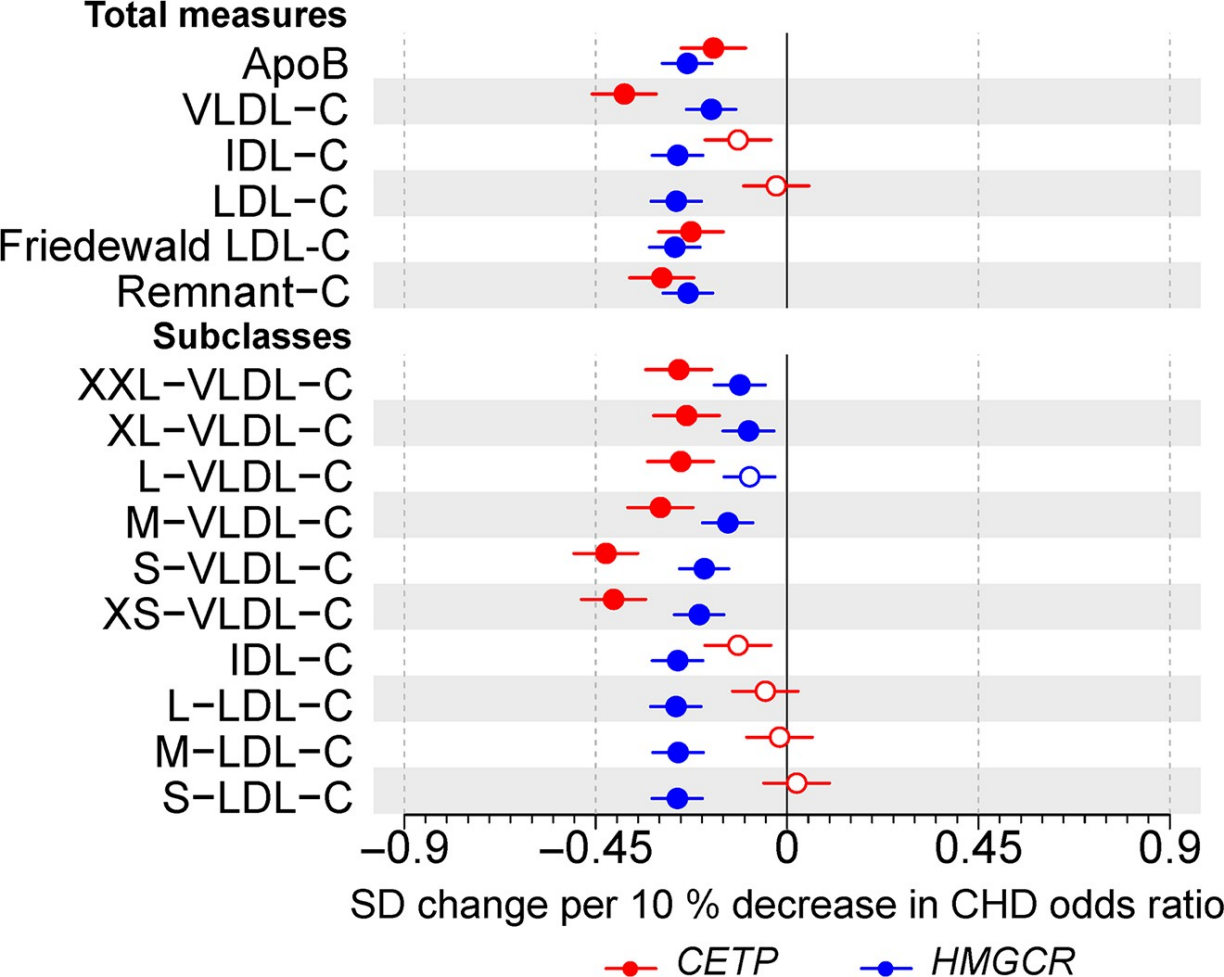
**Associations of genetic variants in *CETP* rs247617 (red) and *HMGCR* rs12916 (blue) with circulating apolipoprotein B and cholesterol concentrations in size-specific apolipoprotein B particles.** Estimates represent the standardized difference in lipoprotein trait, with per allele associations scaled to a 10% lower relative risk of CHD. Analyses were adjusted for age, sex, genotyping batch, and 10 genetic principal components. The circles refer to the effect estimates and the horizontal bars to the 95% CIs. Closed circles represent statistical significance of associations at *P* < 0.002 and open circles associations that are nonsignificant at this threshold. The lipoprotein subclasses are defined by particle size [17–19]: potential chylomicrons and the largest VLDL particles (XXL-VLDL; average particle diameter ≥75 nm); 5 different VLDL subclasses, i.e., very large (average particle diameter 64.0 nm), large (53.6 nm), medium (44.5 nm), small (36.8 nm), and very small VLDL (31.3 nm); IDL (28.6 nm); and 3 LDL subclasses, i.e., large (25.5 nm), medium (23.0 nm), and small LDL (18.7 nm). Underlying data can be found in S1 Data. ApoB, apolipoprotein B; C, cholesterol; CHD, coronary heart disease; IDL, intermediate-density lipoprotein; LDL, low-density lipoprotein; VLDL, very-low-density lipoprotein.

Scaled to 10% reduction in relative risk of CHD, *CETP* rs247617 and *HMGCR* rs12916 had near-identical associations with Friedewald-estimated LDL cholesterol (Fig 1) and similar associations for apolipoprotein B. In contrast, when LDL cholesterol was defined on the basis of cholesterol transported in LDL based on particle size (diameter 18–26 nm) and measured via nuclear magnetic resonance (NMR) spectroscopy, *CETP* expression lowering allele had no association with this size-specific LDL cholesterol (0.02 SDs; 95% CI: −0.10 to 0.05). Although *HMGCR* expression lowering allele had a relatively consistent association with individual apolipoprotein B–containing lipoproteins (effect estimates ranging from −0.25 for intermediate-density lipoprotein [IDL] cholesterol to −0.18 for very-low-density lipoprotein [VLDL] cholesterol), *CETP* expression lowering allele had the most pronounced associations with VLDL cholesterol, a weaker association with IDL cholesterol, but no association with LDL cholesterol defined by particle size or cholesterol transported by any of the large, medium, or small LDL subclasses (Fig 1).

**Fig 2:**
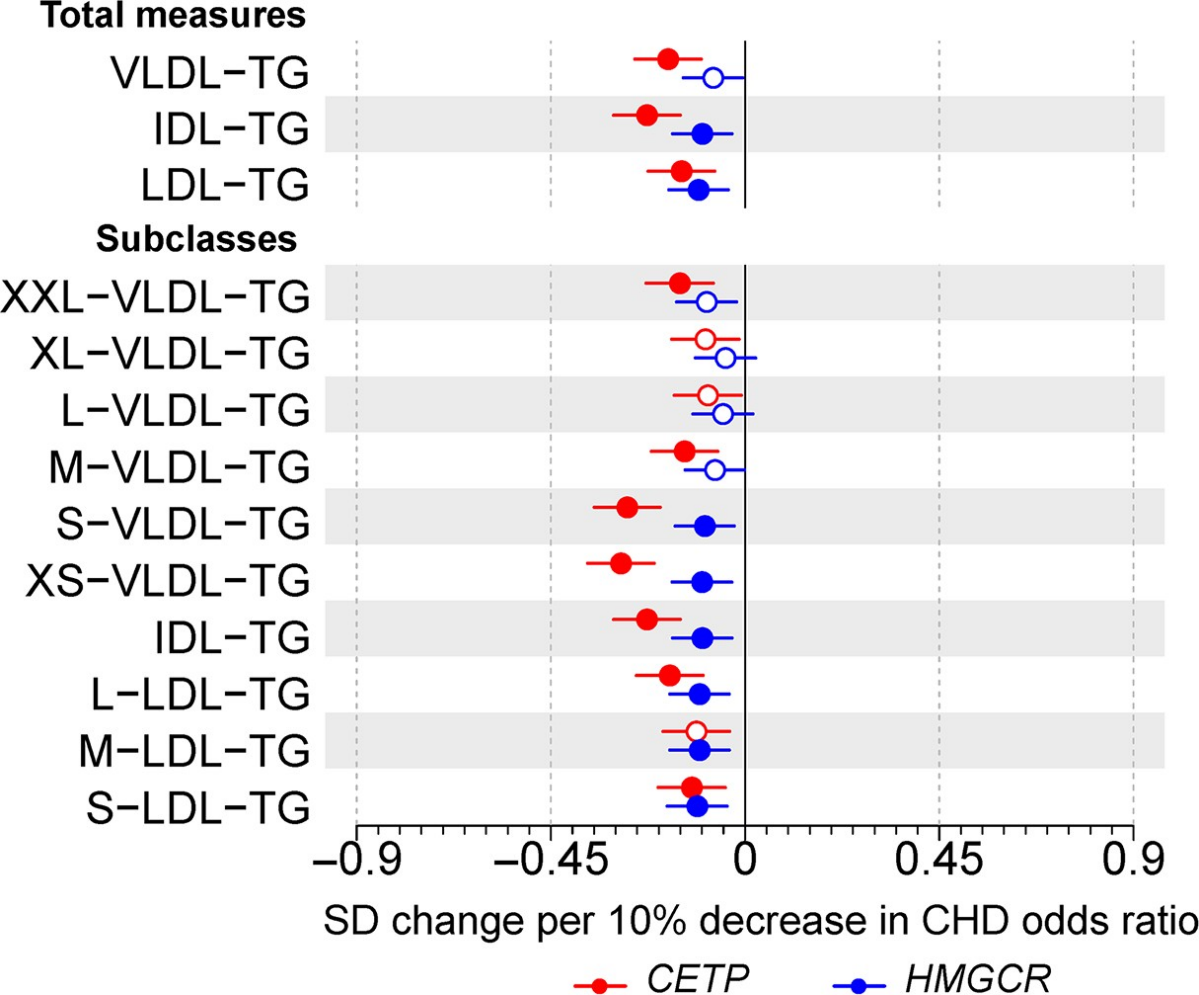
**Associations of genetic variants in *CETP* rs247617 (red) and *HMGCR* rs12916 (blue) with circulating triglyceride concentrations in size-specific apolipoprotein B particles.** Estimates represent the standardized difference in lipoprotein trait, with per allele associations scaled to a 10% lower relative risk of CHD. Analyses were adjusted for age, sex, genotyping batch, and 10 genetic principal components. The circles refer to the effect estimates and the horizontal bars to the 95% CIs. Closed circles represent statistical significance of associations at *P* < 0.002 and open circles associations that are nonsignificant at this threshold. The lipoprotein subclasses are defined by particle size [17–19]: potential chylomicrons and the largest VLDL particles (XXL-VLDL; average particle diameter ≥75 nm); 5 different VLDL subclasses, i.e., very large (average particle diameter 64.0 nm), large (53.6 nm), medium (44.5 nm), small (36.8 nm), and very small VLDL (31.3 nm); IDL (28.6 nm); and 3 LDL subclasses, i.e., large (25.5 nm), medium (23.0 nm), and small LDL (18.7 nm). Underlying data can be found in S1 Data. CHD, coronary heart disease; IDL, intermediate-density lipoprotein; LDL, low-density lipoprotein; TG, triglyceride; VLDL, very-low-density lipoprotein.

**Fig 3:**
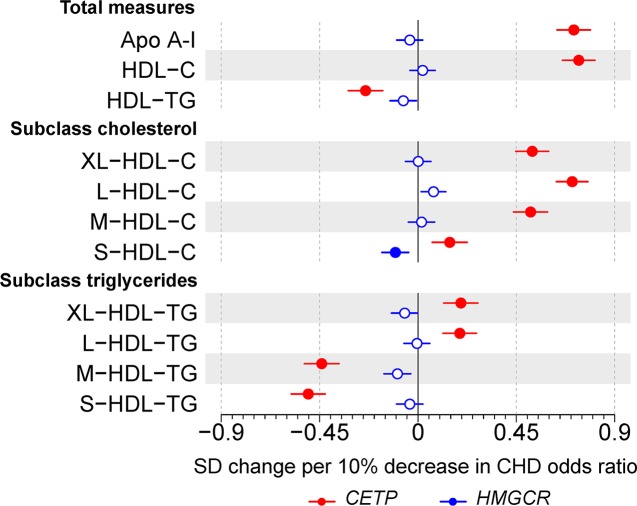
**Associations of genetic variants in *CETP* rs247617 (red) and *HMGCR* rs12916 (blue) with circulating apolipoprotein A-I as well as cholesterol and triglyceride concentrations in size-specific HDL particles.** Estimates represent the standardized difference in lipoprotein trait, with per allele associations scaled to a 10% lower relative risk of CHD. Analyses were adjusted for age, sex, genotyping batch, and 10 genetic principal components. The circles refer to the effect estimates and the horizontal bars to the 95% CIs. Closed circles represent statistical significance of associations at *P* < 0.002 and open circles associations that are nonsignificant at this threshold. The lipoprotein subclasses are defined by particle size [17–19]: the 4 size-specific HDL subclasses are very large (average particle diameter 14.3 nm), large (12.1 nm), medium (10.9 nm), and small HDL (8.7 nm). Underlying data can be found in S1 Data. Apo A-I, apolipoprotein A-I; C, cholesterol; CHD, coronary heart disease; HDL, high-density lipoprotein; TG, triglycerides.

When examining triglycerides in apolipoprotein B–containing particles, *CETP* expression lowering allele associated with lower circulating triglyceride concentrations in VLDL and IDL subclasses, whereas *HMGCR* expression lowering allele had weaker effects on these measures, except in LDL subclasses (Fig 2). *CETP* expression lowering allele had a very strong association with higher HDL cholesterol (0.84; 95% CI: 0.76–0.92) but *HMGCR* did not (0.04; 95% CI: −0.02 to 0.10; Fig 3). Similarly, *CETP* expression lowering allele was associated with lower total quantity of triglycerides in HDL particles (−0.23; 95% CI: −0.31 to −0.15) but *HMGCR* expression lowering allele was not (−0.03; 95% CI: −0.09 to 0.02).

**Fig 4:**
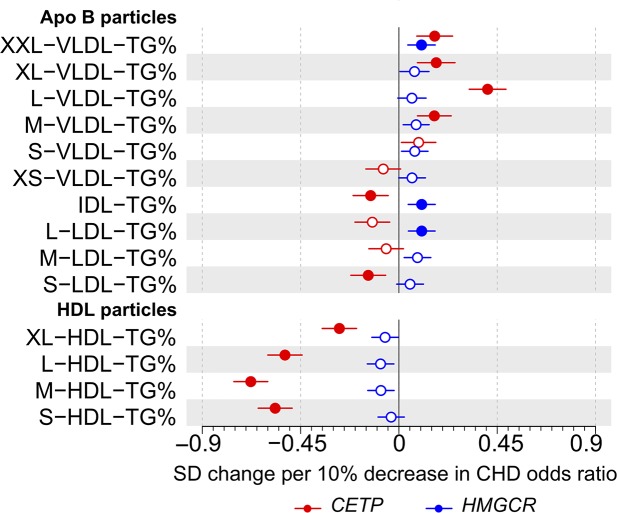
**Associations of genetic variants in *CETP* rs247617 (red) and *HMGCR* rs12916 (blue) with the triglyceride composition of size-specific lipoprotein particles.** Estimates represent the standardized difference in lipoprotein trait, with per allele associations scaled to a 10% lower relative risk of CHD. Analyses were adjusted for age, sex, genotyping batch, and 10 genetic principal components. The circles refer to the effect estimates and the horizontal bars to the 95% CIs. Closed circles represent statistical significance of associations at *P* < 0.002 and open circles associations that are nonsignificant at this threshold. The lipoprotein subclasses are defined by particle size [17–19]: potential chylomicrons and the largest VLDL particles (XXL-VLDL; average particle diameter ≥75 nm); 5 different VLDL subclasses, i.e., very large (average particle diameter 64.0 nm), large (53.6 nm), medium (44.5 nm), small (36.8 nm), and very small (31.3 nm); IDL (28.6 nm); and 3 LDL subclasses, i.e., large (25.5 nm), medium (23.0 nm), and small (18.7 nm). The 4 size-specific HDL subclasses are very large (average particle diameter 14.3 nm), large (12.1 nm), medium (10.9 nm), and small (8.7 nm). Underlying data can be found in S1 Data. Apo B, apolipoprotein B; CHD, coronary heart disease; HDL, high-density lipoprotein; IDL, intermediate-density lipoprotein; LDL, low-density lipoprotein; TG, triglycerides; VLDL, very-low-density lipoprotein.

The lipoprotein particle structure is biophysically constrained, generating strong correlations between lipid measures within individual lipoprotein subclasses [19–22]. Notable differences in lipid concentrations in subclass particles would therefore suggest changes in the compositional proportions of these lipids. For genetic inhibition of CETP, the effects on circulating triglyceride concentrations in all HDL subclasses were weaker (XL-HDL and L-HDL) or even in the opposite direction (M-HDL and S-HDL) than the effects on cholesterol concentration in these subclasses (Fig 3). Examining the genetic associations with the particle lipid compositions, the relative amount of triglycerides (in relation to all lipid molecules in the particles) was remarkably diminished in all HDL subclass particles by genetic inhibition of CETP (Fig 4). Genetic inhibition of HMGCR did not associate with triglyceride concentration or composition of any HDL subclass. These associations are in line with the known physiological roles of CETP and HMGCR and their inhibition [23,24]. In addition, as expected, *CETP* expression lowering allele associated with higher compositions of triglycerides in most VLDL subclass particles and *HMGCR* expression lowering allele showed directionally similar albeit weaker associations.

**Fig 5:**
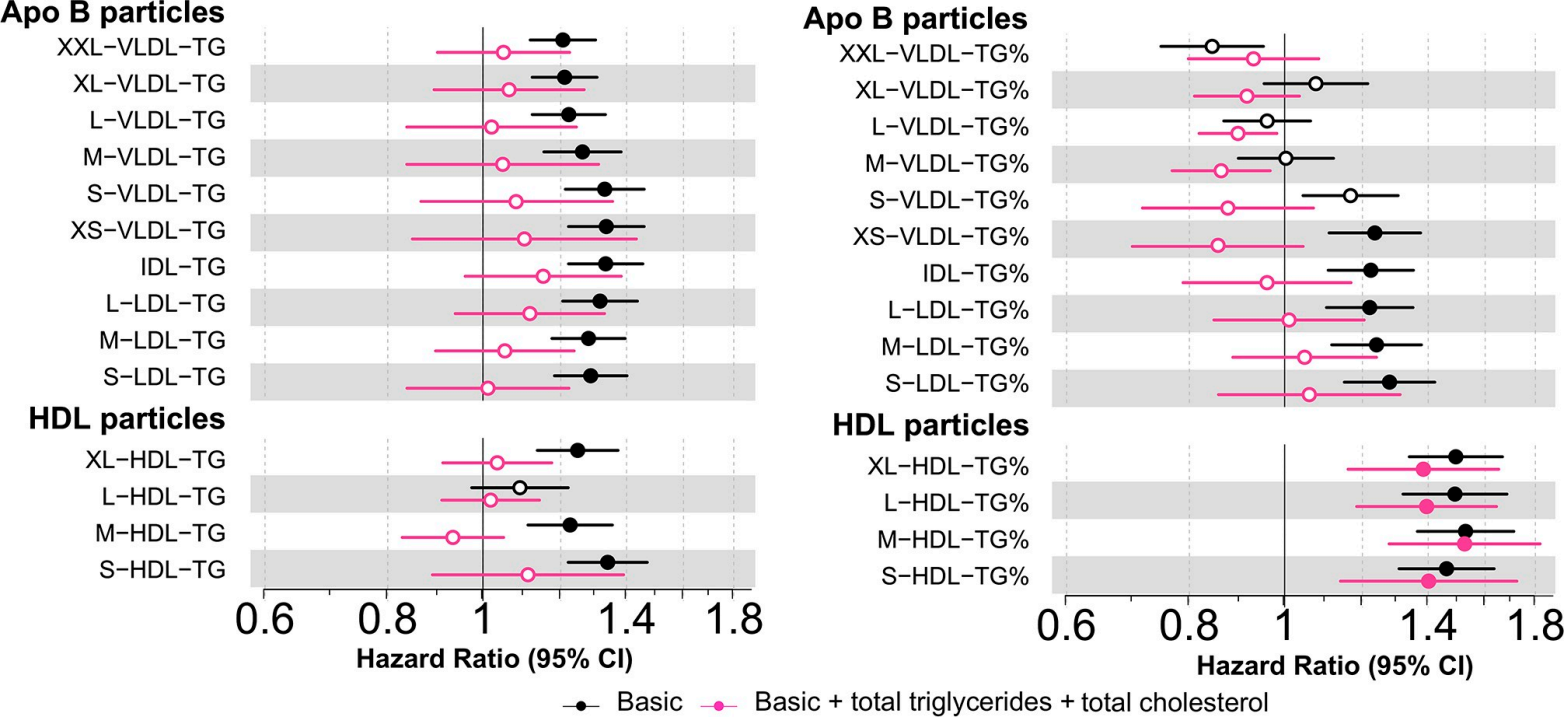
Observational associations of circulating triglyceride concentrations and triglyceride composition in lipoprotein subclass particles and risk of incident CHD. (Left panel) Black: Hazard ratios for incident CHD per SD higher triglyceride concentration within each size-specific lipoprotein subclass adjusted for traditional risk factors. Pink: adjusted for traditional risk factors, serum cholesterol, and serum triglycerides. (Right panel) Black: Hazard ratios for incident CHD per SD higher percentage of triglycerides (of all lipid molecules) within each size-specific lipoprotein subclass adjusted for traditional risk factors. Pink: adjusted for traditional risk factors, serum cholesterol, and serum triglycerides. Basic risk factors include age, sex, mean arterial pressure, smoking, diabetes mellitus, lipid medication, geographical region in FINRISK, and ethnicity in SABRE. The horizontal bars to the 95% CIs. Closed circles represent statistical significance of associations at *P* < 0.002 and open circles associations that are nonsignificant at this threshold. The horizontal bars refer to the 95% CIs. Underlying data can be found in S1 Data. Apo B, apolipoprotein B; CHD, coronary heart disease; HDL, high-density lipoprotein; IDL, intermediate-density lipoprotein; LDL, low-density lipoprotein; VLDL, very-low-density lipoprotein; TG, triglycerides.

To understand the clinical relevance of these HDL-related compositional changes arising from CETP inhibition, beyond lowering the cholesterol concentrations of apolipoprotein B–containing lipoprotein particles, we studied the observational associations of lipoprotein subclass lipid concentrations and compositions with CHD in 3 prospective population cohorts totalling 616 incident cases and 13,564 controls during an 8-year follow-up. The triglyceride concentration of HDL was associated with incident CHD when adjusted for nonlipid cardiovascular risk factors (Fig 5). However, when serum cholesterol and serum triglycerides were added to the model, as expected, the associations attenuated. In contrast, the triglyceride compositions of all the HDL subclass particles were positively associated with CHD, independent of circulating concentrations of cholesterol and triglycerides, with hazard ratios around 1.3 for all HDL subclasses (Fig 5). Adjusting for LDL-C had only very minor effects on the associations of both circulating HDL-related triglyceride concentrations and the triglyceride compositions of HDL particles. In addition to the compositional enrichment of triglycerides in HDL particles, the compositional enrichment of cholesteryl esters in the largest VLDL particles (XXL-VLDL and XL-VLDL) was also observationally associated with greater risk of CHD (S11 Fig). The genetic inhibition of CETP lowered the cholesteryl ester composition of these VLDL particles, i.e., was acting toward decreased risk of CHD (S2 Fig).

**Fig 6:**
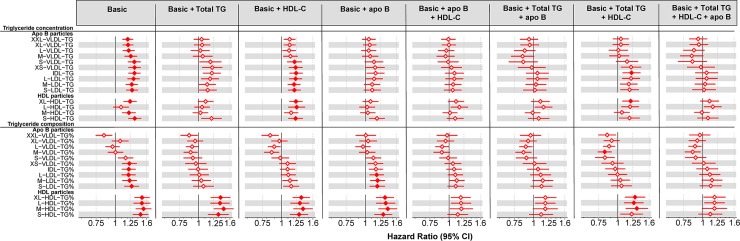
Observational associations of circulating triglyceride concentrations and triglyceride composition in lipoprotein subclass particles and risk of incident CHD with multiple adjustments. Hazard ratios for incident CHD per SD higher circulating triglyceride concentrations (upper part) and triglyceride composition (lower part) in lipoprotein subclass particles within each size-specific lipoprotein subclass adjusted for traditional risk factors and gradually for 3 fundamental measures that characterize the overall lipoprotein profile pretty well, namely, total serum triglyceride (total TG), HDL cholesterol (HDL-C), and apolipoprotein B (apoB) concentration. Closed circles represent statistical significance of associations at *P* < 0.002 and open circles associations that are nonsignificant at this threshold. The horizontal bars refer to the 95% CIs. Underlying data can be found in S1 Data. Apo B, apolipoprotein B; C, cholesterol; CHD, coronary heart disease; HDL, high-density lipoprotein; IDL, intermediate-density lipoprotein; LDL, low-density lipoprotein; TG, triglycerides; VLDL, very-low-density lipoprotein.

To further investigate the novel relation of the triglyceride composition of HDL particles (and thereby potentially the inhibition of CETP) with incident CHD, we performed systematic analyses focusing on 3 fundamental measures that characterize the overall lipoprotein profile fairly well, namely, total serum triglyceride, HDL-C, and apolipoprotein B concentration, and gradually adjusted the association between HDL particle triglyceride composition and incident CHD. The results are presented in Fig 6. Adjusting the associations between HDL particle triglyceride compositions and incident CHD with total triglycerides, HDL-C and apolipoprotein B had all very similar minor effects. However, a combined adjustment for apolipoprotein B and HDL-C almost abolished the associations similarly to apolipoprotein B and triglycerides.

## Discussion

We used genetic variants in *CETP* and *HMGCR* to gain insight into the expected effects of therapeutic inhibition of CETP and HMG-CoA reductase on circulating lipoproteins and lipids. Our data show that although *CETP* and *HMGCR* have near-identical effects on Friedewald-estimated LDL cholesterol, this result masks a very different association of *CETP* and *HMGCR* with size-specific LDL cholesterol. Genetic inhibition of HMGCR showed similar effects with cholesterol across the apolipoprotein B–containing lipoproteins but genetic inhibition of CETP showed stronger associations with larger apolipoprotein B particles, namely, VLDL and remnant cholesterol [25], but no association with cholesterol carried specifically in LDL particles defined by size.

Friedewald-estimated LDL cholesterol (as well as ‘direct’ assays) are nonspecific measures of cholesterol [26–28]. For example, in addition to the cholesterol in size-specific LDL particles, Friedewald LDL cholesterol also includes, to varying degrees, cholesterol in IDL, VLDL, and lipoprotein(a) [29]. This nonspecificity of commonly used “LDL” cholesterol assays is under-recognized and underlies the prevailing opinion that inhibitors of HMGCR and CETP both alter LDL cholesterol. However, our data show this not to be the case: using NMR spectroscopy-based lipoprotein particle quantification, which defines individual lipoprotein subclasses based on particle size [18,19,21], our findings demonstrate that CETP has negligible effect on cholesterol in size-specific LDL particles. As the inhibition of CETP affects the IDL subclass similarly to all the LDL subclasses, the “LDL cholesterol” via beta-quantification would also be only minimally affected. In this way, the use of a composite lipid measure can obscure differential associations of a therapy or gene [20] with individual constituents of the composite and can have clinical ramifications. For example, if a trial is powered to a given reduction in Friedewald LDL cholesterol, under the naïve assumption that the drug uniformly alters all the subcomponents, then the trial may not have the expected result if the drug has differential effects on these subcomponents. This is exemplified in the recent phase III ACCELERATE trial of evacetrapib, which was terminated for futility, and was powered to a difference in LDL cholesterol based on a composite assay [12]. The differential effects of CETP inhibition on composite markers such as Friedewald and directly-quantified LDL cholesterol compared to apolipoprotein B concentrations identified in the subsequent phase III REVEAL trial of anacetrapib [13] suggest that had ACCELERATE used an alternative measure of proatherogenic lipoproteins (e.g., apolipoprotein B or non-HDL-C [14]) to gauge the expected vascular effect, the trial may have been more appropriately powered.

This highlights the need to understand, in detail, the consequences of lipid-modifying therapies on lipoproteins and lipids in order to be able to gauge whether a composite measure (such as Friedewald LDL cholesterol) can be reliably used as an indicator of the likely beneficial effect of a therapy. This is unlikely to be limited to assays for LDL cholesterol. For example, assays that quantify triglycerides measure the summation of triglycerides across multiple lipoprotein particle categories. Drugs currently under development that target triglycerides (such as apolipoprotein C-III inhibitors [30]) have differential effects on triglycerides in lipoprotein subclass particles as demonstrated in a recent genetic study [31]. If triglycerides within different lipoprotein subclasses have heterogeneous effects on vascular disease, a clinical trial powered to the overall concentration of circulating triglycerides may give an inaccurate portrayal of the cardiovascular consequences arising from apolipoprotein C-III inhibition.

Another key finding is that the lipid compositions of lipoprotein particles can associate with disease risk independently of total lipid concentrations. Although genetic inhibition of CETP increased circulating concentrations of cholesterol in all HDL subclasses, the triglyceride composition, i.e., the percentage of triglyceride molecules of all the lipid molecules in the particle, was markedly lower in all HDL particles. Intriguingly, our observational analyses, the first to explore lipoprotein particle lipid composition with CHD outcomes, revealed that triglyceride enrichment of HDL particles associates with higher risk for future CHD, independently of total circulating cholesterol and triglycerides. The largest hazard ratio for the triglyceride enrichment in medium HDL subclass particles was of a similar magnitude (approximately 1.3) as that for LDL cholesterol and apolipoprotein B [32]. However, this phenomenon appears to be due to combined effects of circulating HDL and apolipoprotein B-containing particles, maybe in connection to CETP function and the circulating amount of total triglycerides, not an intrinsic indication of the role of HDL particle lipid composition in CHD.

Key strengths of our analyses include the availability of detailed measurements of blood lipoprotein subclass concentrations and compositions from general population studies with incident CHD events, together with the availability of genome-wide genotyping. We used single *CETP* and *HMGCR* variants as genetic proxies for therapeutic inhibition (i.e., instruments in the Mendelian randomization analyses), assuming that they are not pleiotropic. This assumption is justifiable on the basis that the SNPs were selected in *cis*-regions and alter gene expression and together with the fact that (1) the *CETP* genetic variant recapitulated the effects of CETP enzyme activity in relation to the role the enzyme has in shuttling esterified cholesterol from HDL to apolipoprotein B-containing particles in exchange for triglycerides [23] and that (2) prospective population-based data of patients taking statins with blood sampling before and after the commencement of therapy showed that genetic variants in *HMGCR* robustly recapitulated the effects of statin therapy on lipoprotein subclasses and lipids [16].

In conclusion, we have shown that, in contrast to genetic inhibition of HMG-CoA (proxying statin therapy), genetic inhibition of CETP does not alter circulating size-specific LDL cholesterol concentrations. This is masked by using conventional, nonspecific assays for LDL cholesterol and may be problematic for ongoing and future clinical trials of lipid lowering therapies, especially when a nonspecific marker of lipids is used to derive an expected effect of a drug with risk of disease. The basis for the reduction in CHD risk seen with CETP inhibition appears to be due to the lowering of atherogenic non-LDL lipoprotein particles. Our findings draw attention to the need for metabolic precision in measurements of lipoprotein lipids and subclasses and in assessing the role of lipoprotein metabolism in cardiovascular disease in relation to ongoing treatment trials of novel lipid-altering therapies.

## Methods

### Ethics statement

The Ethics Committee of the Faculty of Medicine, University of Oulu has approved the Northern Finland Birth Cohort 1986 (NFBC86) (17.6.1999) and the Northern Finland Birth Cohort 1966 (NFBC66) studies (17.6.1996). In addition, the Ethics Committee of the Northern Ostrobothnia Hospital District has approved the NFBC66 (94/2011) and NFBC86 (108/2017). This study has been approved by the NFBC Scientific Committee (material request P0268/2018). The Cardiovascular Risk in Young Finns Study (YFS) was approved by the following Ethics Committees covering all the 5 participating medical university study sites in Finland: the Ethics Committee of the Hospital District of Southwest Finland (12/2007 §533, 19.12.2006; 8/2007 §330, 28.8.2007; 1/2008 §28, 15.1.2008), the Ethics Committee of the Pirkanmaa Hospital District (ETL-R07100), and the Ethics Committee of the Northern Ostrobothnia Hospital District (84/2001). The FINRISK 1997 was approved by the Ethics committee of the National Public Health Institute, Helsinki, Finland (23.01.1997), and the DILGOM 2007 study was approved by the Ethics Committee of the Helsinki and Uusimaa Hospital District (229/E0/2006). The SABRE study protocols were approved by the University College London (5.1.1988/PMcK/sp) and by the St. Mary’s Hospital Research Ethics Committee (07/H0712/109). The INTERVAL study was approved by the Cambridge (East) Research Ethics Committee (11/EE/0538/74247) and was also approved by the University of Cambridge’s Research Operations Office and the Research Governance Office. All studies were approved by local institutional research review committees, and all clinical investigations were conducted according to the principles expressed in the Declaration of Helsinki. All participants gave written informed consents.

### Prospective and cross-sectional studies and lipoprotein quantification

We used genetic and lipoprotein data from 5 population-based Finnish cohorts and 1 cross-sectional study in the UK (cohort characteristics are presented in S1 Table and study descriptions are given in S1 Text). Details of study-specific genotyping are provided in S2 Table. Briefly, the cohorts used were the NFBC66 (*n* = 4,702 individuals aged 31 y at blood draw) [33,34], the NFBC86 (*n* = 3,726 individuals aged 16 y at blood draw), the YFS (*n* = 1,948 individuals aged 24–39 y in 2007) [35], 2 population-based Finnish cohorts FINRISK 1997 (*n* = 6,942 individuals aged 24–74 y) and DILGOM subsample of FINRISK 2007 (*n* = 4,124 individuals aged 24–74 y) [36,37], and a study of healthy blood donors from the UK (INTERVAL: *n* = 40,958 individuals aged 18–80 y) [38]. For prospective analyses, we used the abovementioned FINRISK 1997 and DILGOM cohorts and additionally a tri-ethnic UK community-based cohort SABRE (*n* = 4,976 individuals aged 40–69 y) [39,40]. The focus in this study was to evaluate the impact of variants in *CETP* (and *HMGCR*) on lipoprotein metabolism, i.e., on the entire cascade of apolipoprotein B–containing lipoproteins and HDL subclasses. Therefore, we decided a priori to examine all the 191 lipoprotein and lipid traits available from the NMR-based methodology [17]. Abbreviations and full descriptions of the lipoprotein measures are given in S3 Table. Details of this platform have been published previously [17,41], and it has been widely applied in genetic and epidemiological studies [16,18,42–44]. Focusing on these 191 traits, we estimated that 28 principal components explain 99% of their variation in the Finnish cohorts, and therefore we used a *P* value threshold of 0.05/28 = 0.002 to denote evidence in favor of an association.

Where possible, we excluded individuals receiving lipid lowering medication, pregnant women, and those who had a high proportion (>30%) of values missing across the lipid traits; details are given in S1 Text. All measures (S3 Table) were first adjusted for sex, age (if applicable), genotyping batch (if applicable), and 10 first principal components from genomic data, and the resulting residuals were transformed to normal distribution by inverse rank-based normal transformation.

### Selection of genetic variants and genetic analyses

We selected variants as genetic proxies of CETP and HMGCR inhibition on the basis of robust associations with circulating lipids in GWAS consortia [42,45] and target gene expression. The *HMGCR* variant (rs12916) LDL cholesterol lowering T allele (−0.24 SD LDL cholesterol per T allele; *P* = 1.3 × 10^−14^) has been shown to lower *HMGCR* expression [46,47], and the *CETP* variant (rs247617) HDL cholesterol increasing A allele (0.84 SD HDL cholesterol per A allele; P = 5.4 × 10^−94^) associates with lower *CETP* gene expression. Rs247617 is the strongest eQTL for CETP across all tissues in Genotype To Expression (https://gtexportal.org) project data [48]. Thus, we use these variants as biologically plausible instruments in the Mendelian randomization framework to infer the drug effects through genetic inhibition of these genes [6,33]. We used an additive model for each cohort separately (see S1 Table for details of analysis software). In order to make the lipoprotein and lipid estimates comparable, the estimates for *CETP* rs247617 and *HMGCR* rs12916 were scaled to the same CHD association as reported by the CARDIoGRAMplusC4D GWAS Consortium [49]. The per allele log odds (logOR) for CHD was 0.0358 (standard error = 0.01, P = 1.6 × 10^−4^ and T allele frequency 0.57) for *HMGCR* rs12916 and 0.0309 (standard error = 0.01, P = 2.5 × 10^−3^ and C allele frequency 0.69) for *CETP* rs247617; subsequently, the summary statistics of each individual cohort and each metabolite were scaled to −0.105 logOR of CHD (equivalent to an odds ratio [OR] of CHD of 0.90) to align the estimates to a 10% lower relative risk of CHD. We use the term relative risk as a moniker of ratio effect estimates. The cohort-specific association results of lipoprotein and lipid measures with both variants were then combined using an inverse-variance weighted fixed effect meta-analysis.

### Association of lipoprotein measures with risk of incident CHD

Cohorts contributing to the associations of lipoprotein lipid concentration and composition measures and the hazard of incident CHD were FINRISK 1997, DILGOM, and SABRE. Participants with prevalent CHD were excluded from the analysis. Following exclusion, data were available from FINRISK 1997 for 6,484 individuals (287 cases/6,197 controls) and 3,318 individuals from DILGOM (270 cases/3,048 controls) and for SABRE 4,378 individuals with nonmissing data (59 cases/4,319 controls). The follow-up time of FINRISK 1997 and SABRE were censored to 8 years to match the follow-up time in DILGOM.

Prior to statistical analyses, metabolic measures were log-transformed and scaled to SD in each cohort. The relationships of lipid measures with the risk of CHD were analysed using Cox proportional hazards regression models with age, sex, mean arterial pressure, smoking, diabetes mellitus, lipid medication, and geographical region (Finnish cohorts), ethnicity (SABRE), total cholesterol, and total triglyceride concentrations as covariates. The cohort-specific association results of 191 lipid measures were then combined using inverse-variance weighted fixed-effects meta-analysis. Analyses were conducted in R studio (version 1.0.153, R version 3.3.3). As above, we used a *P* value threshold of ≤0.002 to denote evidence in favor of an association.

## Supporting information

S1 Text Study descriptions. Overall description of the individual cohorts.
(PDF)

S1 Table Characteristics of the study populations. Clinical characteristics for the 7 cohorts included in the study.
(PDF)

S2 Table Genotyping detail of the cohorts. Details of the genotyping for the 6 cohorts including genetic data in this study.
(PDF)

S3 Table Key for the lipid and lipoprotein abbreviations. Abbreviations and the description of the lipid and lipoprotein subclass measures.
(PDF)

S1 Fig **Meta-analysis of genetic variants in *CETP* rs247617 (red) and *HMGCR* rs12916 (blue) for all lipoprotein concentration measures.** Estimates are the standardized difference in lipoprotein trait, with per allele associations scaled to a 10% lower relative risk of CHD. The circles refer to the effect estimates and the horizontal bars to the 95% CIs. Closed circles represent associations at *P* < 0.002, and open circles show associations that are nonsignificant at this threshold. The lipoprotein subclasses are defined by particle size: potential chylomicrons and the largest VLDL particles (XXL-VLDL; average particle diameter *≥*75 nm); 5 different VLDL subclasses, i.e., very large (average particle diameter 64.0 nm), large (53.6 nm), medium (44.5 nm), small (36.8 nm), and very small (31.3 nm); IDL (28.6 nm); and 3 LDL subclasses, i.e., large (25.5 nm), medium (23.0 nm), and small (18.7 nm). Underlying data can be found in S2 Data. CHD, coronary heart disease; IDL, intermediate-density lipoprotein; LDL, low-density lipoprotein; VLDL, very-low-density lipoprotein.
(TIF)

S2 Fig **Meta-analysis of genetic variants in *CETP* rs247617 (red) and *HMGCR* rs12916 (blue) for all lipoprotein composition measures.** Estimates are the standardized difference in lipoprotein trait, with per allele associations scaled to a 10% lower relative risk of CHD. The circles refer to the effect estimates and the horizontal bars to the 95% CIs. Closed circles represent associations at *P* < 0.002, and open circles show associations that are nonsignificant at this threshold. The lipoprotein subclasses are defined by particle size: potential chylomicrons and the largest VLDL particles (XXL-VLDL; average particle diameter *≥*75 nm); 5 different VLDL subclasses, i.e., very large (average particle diameter 64.0 nm), large (53.6 nm), medium (44.5 nm), small (36.8 nm), and very small (31.3 nm); IDL (28.6 nm); and 3 LDL subclasses, i.e., large (25.5 nm), medium (23.0 nm), and small (18.7 nm). Underlying data can be found in S2 Data. CHD, coronary heart disease; IDL, intermediate-density lipoprotein; LDL, low-density lipoprotein; VLDL, very-low-density lipoprotein.
(TIF)

S3 Fig **Meta-analysis of genetic variants in *CETP* rs247617 (red) and *HMGCR* rs12916 (blue) for all summary lipid measures.** Estimates are the standardized difference in lipoprotein trait, with per allele associations scaled to a 10% lower relative risk of CHD. The circles refer to the effect estimates and the horizontal bars to the 95% CIs. Closed circles represent associations at *P* < 0.002, and open circles show associations that are nonsignificant at this threshold. The lipoprotein subclasses are defined by particle size: potential chylomicrons and the largest VLDL particles (XXL-VLDL; average particle diameter *≥*75 nm); 5 different VLDL subclasses, i.e., very large (average particle diameter 64.0 nm), large (53.6 nm), medium (44.5 nm), small (36.8 nm), and very small (31.3 nm); IDL (28.6 nm); and 3 LDL subclasses, i.e., large (25.5 nm), medium (23.0 nm), and small LDL (18.7 nm). Underlying data can be found in S2 Data. CHD, coronary heart disease; IDL, intermediate-density lipoprotein; LDL, low-density lipoprotein; VLDL, very-low-density lipoprotein.
(TIF)

S4 Fig Individual cohort associations of genetic variant in *CETP* rs247617 for all lipoprotein concentration measures. Estimates are the standardized difference in lipoprotein trait, with per allele associations scaled to a 10% lower relative risk of CHD. The circles refer to the effect estimates and the horizontal bars to the 95% CIs. Closed circles represent associations at *P* < 0.002, and open circles show associations that are nonsignificant at this threshold. The lipoprotein subclasses are defined by particle size: potential chylomicrons and the largest VLDL particles (XXL-VLDL; average particle diameter *≥*75 nm); 5 different VLDL subclasses, i.e., very large (average particle diameter 64.0 nm), large (53.6 nm), medium (44.5 nm), small (36.8 nm), and very small (31.3 nm); IDL (28.6 nm); and 3 LDL subclasses, i.e., large (25.5 nm), medium (23.0 nm), and small (18.7 nm). Underlying data can be found in S2 Data. CHD, coronary heart disease; IDL, intermediate-density lipoprotein; LDL, low-density lipoprotein; VLDL, very-low-density lipoprotein.
(TIF)

S5 Fig Individual cohort results of *CETP* rs247617 for all lipoprotein composition measures. Estimates are the standardized difference in lipoprotein trait, with per allele associations scaled to a 10% lower relative risk of CHD. The circles refer to the effect estimates and the horizontal bars to the 95% CIs. Closed circles represent associations at *P* < 0.002, and open circles show associations that are nonsignificant at this threshold. The lipoprotein subclasses are defined by particle size: potential chylomicrons and the largest VLDL particles (XXL-VLDL; average particle diameter *≥*75 nm); 5 different VLDL subclasses, i.e., very large (average particle diameter 64.0 nm), large (53.6 nm), medium (44.5 nm), small (36.8 nm), and very small (31.3 nm); IDL (28.6 nm); and 3 LDL subclasses, i.e., large (25.5 nm), medium (23.0 nm), and small (18.7 nm). Underlying data can be found in S2 Data. CHD, coronary heart disease; IDL, intermediate-density lipoprotein; LDL, low-density lipoprotein; VLDL, very-low-density lipoprotein.
(TIF)

S6 Fig Individual cohort associations of *CETP* rs247617 for all summary lipid measures. Estimates are the standardized difference in lipoprotein trait, with per allele associations scaled to a 10% lower relative risk of CHD. The circles refer to the effect estimates and the horizontal bars to the 95% CIs. Closed circles represent associations at *P* < 0.002, and open circles show associations that are nonsignificant at this threshold. The lipoprotein subclasses are defined by particle size: potential chylomicrons and the largest VLDL particles (XXL-VLDL; average particle diameter *≥*75 nm); 5 different VLDL subclasses, i.e., very large (average particle diameter 64.0 nm), large (53.6 nm), medium (44.5 nm), small (36.8 nm), and very small (31.3 nm); IDL (28.6 nm); and 3 LDL subclasses, i.e., large (25.5 nm), medium (23.0 nm), and small (18.7 nm). Underlying data can be found in S2 Data. CHD, coronary heart disease; IDL, intermediate-density lipoprotein; LDL, low-density lipoprotein; VLDL, very-low-density lipoprotein.
(TIF)

S7 Fig Individual cohort association of *HMGCR* rs12916 variant for all lipoprotein concentration measures. Estimates are the standardized difference in lipoprotein trait, with per-allele associations scaled to a 10% lower relative risk of CHD. The circles refer to the effect estimates and the horizontal bars to the 95% CIs. Closed circles represent associations at *P* < 0.002, and open circles show associations that are nonsignificant at this threshold. The lipoprotein subclasses are defined by particle size: potential chylomicrons and the largest VLDL particles (XXL-VLDL; average particle diameter *≥*75 nm); 5 different VLDL subclasses, i.e., very large (average particle diameter 64.0 nm), large (53.6 nm), medium (44.5 nm), small (36.8 nm), and very small (31.3 nm); IDL (28.6 nm); and 3 LDL subclasses, i.e., large (25.5 nm), medium (23.0 nm), and small (18.7 nm). Underlying data can be found in S2 Data. CHD, coronary heart disease; IDL, intermediate-density lipoprotein; LDL, low-density lipoprotein; VLDL, very-low-density lipoprotein.
(TIF)

S8 Fig Individual cohort results of *HMGCR* rs12916 association with all lipoprotein composition measures. Estimates are the standardized difference in lipoprotein trait, with per allele associations scaled to a 10% lower relative risk of CHD. The circles refer to the effect estimates and the horizontal bars to the 95% CIs. Closed circles represent associations at *P* < 0.002, and open circles show associations that are nonsignificant at this threshold. The lipoprotein subclasses are defined by particle size: potential chylomicrons and the largest VLDL particles (XXL-VLDL; average particle diameter *≥*75 nm); 5 different VLDL subclasses, i.e., very large (average particle diameter 64.0 nm), large (53.6 nm), medium (44.5 nm), small (36.8 nm), and very small (31.3 nm); IDL (28.6 nm); and 3 LDL subclasses, i.e., large (25.5 nm), medium (23.0 nm), and small (18.7 nm). Underlying data can be found in S2 Data. CHD, coronary heart disease; IDL, intermediate-density lipoprotein; LDL, low-density lipoprotein; VLDL, very-low-density lipoprotein.
(TIF)

S9 Fig Individual cohort results of *HMGCR* rs12916 associations with all summary lipid measures. Estimates are the standardized difference in lipoprotein trait, with per allele associations scaled to a 10% lower relative risk of CHD. The circles refer to the effect estimates and the horizontal bars to the 95% CIs. Closed circles represent associations at *P* < 0.002, and open circles show associations that are nonsignificant at this threshold. The lipoprotein subclasses are defined by particle size: potential chylomicrons and the largest VLDL particles (XXL-VLDL; average particle diameter *≥*75 nm); 5 different VLDL subclasses, i.e., very large (average particle diameter 64.0 nm), large (53.6 nm), medium (44.5 nm), small (36.8 nm), and very small (31.3 nm); IDL; 28.6 nm); and 3 LDL subclasses, i.e., large (25.5 nm), medium (23.0 nm), and small (18.7 nm). Underlying data can be found in S2 Data. CHD, coronary heart disease; IDL, intermediate-density lipoprotein; LDL, low-density lipoprotein; VLDL, very-low-density lipoprotein.
(TIF)

S10 Fig Meta-analysis of incident CHD association for all lipoprotein concentration measures. Estimates represent hazard ratios for incident CHD per SD lipoprotein concentration. Black color refers to adjusting for the traditional risk factors and pink color adjusting for the traditional risk factors and serum cholesterol and serum triglycerides. Traditional risk factors include age, sex, mean arterial pressure, smoking, diabetes mellitus, lipid medication, geographical region in FINRISK, and ethnicity in SABRE. Closed circles represent statistical significance of associations at *P* < 0.002 and open circles associations that are nonsignificant at this threshold. The horizontal bars refer to the 95% CIs. Underlying data can be found in S2 Data. CHD, coronary heart disease.
(TIF)

S11 Fig Meta-analysis of incident CHD association for all lipoprotein composition measures. Estimates represent hazard ratios for incident CHD per SD lipoprotein composition measure. Black color refers to adjusting for the traditional risk factors and pink color adjusting for the traditional risk factors and serum cholesterol and serum triglycerides. Traditional risk factors include age, sex, mean arterial pressure, smoking, diabetes mellitus, lipid medication, geographical region in FINRISK, and ethnicity in SABRE. Closed circles represent statistical significance of associations at *P* < 0.002 and open circles associations that are nonsignificant at this threshold. The horizontal bars refer to the 95% CIs. Underlying data can be found in S2 Data. CHD, coronary heart disease.
(TIF)

S12 Fig Meta-analysis of incident CHD association for all summary lipid measures. Estimates represent hazard ratios for incident CHD per SD lipoprotein measure. Black colo r refers to adjusting for the traditional risk factors and pink color adjusting for the traditional risk factors and serum cholesterol and serum triglycerides. Traditional risk factors include age, sex, mean arterial pressure, smoking, diabetes mellitus, lipid medication, geographical region in FINRISK, and ethnicity in SABRE. Closed circles represent statistical significance of associations at *P* < 0.002 and open circles associations that are nonsignificant at this threshold. The horizontal bars refer to the 95% CIs. Underlying data can be found in S2 Data. CHD, coronary heart disease.
(TIF)

S13 Fig Individual cohort results of incident CHD association for all lipoprotein concentration measures. Estimates represent hazard ratios for incident CHD per SD lipoprotein concentration adjusted for traditional risk factors (age, sex, mean arterial pressure, smoking, diabetes mellitus, lipid medication, geographical region in FINRISK, and ethnicity in SABRE), total cholesterol, and total triglycerides. Closed circles represent statistical significance of associations at *P* < 0.002 and open circles associations that are nonsignificant at this threshold. The horizontal bars refer to the 95% CIs. Underlying data can be found in S2 Data. CHD, coronary heart disease.
(TIF)

S14 Fig Individual cohort results of incident CHD association for all lipoprotein composition measures. Estimates represent hazard ratios for incident CHD per SD lipoprotein composition measure adjusted for traditional risk factors (age, sex, mean arterial pressure, smoking, diabetes mellitus, lipid medication, geographical region in FINRISK, and ethnicity in SABRE), total cholesterol, and total triglycerides. Closed circles represent statistical significance of associations at *P* < 0.002 and open circles associations that are nonsignificant at this threshold. The horizontal bars refer to the 95% CIs. Underlying data can be found in S2 Data. CHD, coronary heart disease.
(TIF)

S15 Fig Individual cohort results of incident CHD association for all summary lipid measures. Estimates represent hazard ratios for incident CHD per SD lipoprotein measure adjusted for traditional risk factors (age, sex, mean arterial pressure, smoking, diabetes mellitus, lipid medication, geographical region in FINRISK, and ethnicity in SABRE), total cholesterol, and total triglycerides. Closed circles represent statistical significance of associations at *P* < 0.002 and open circles associations that are nonsignificant at this threshold. The horizontal bars refer to the 95% CIs. Underlying data can be found in S2 Data. CHD, coronary heart disease.
(TIF)

S1 Data Data underlying Figs 1–6. (XLSX)

S2 Data Data underlying S1–S15 Figs. (XLSX)

## Abbreviations

Apo A-I: apolipoprotein A-I
ApoB: apolipoprotein B
C: cholesterol
CETP: cholesteryl ester transfer protein
CHD: coronary heart disease
CoA: coenzyme A
HDL: high-density lipoprotein
HMGCR: 3-hydroxy-3-methyl-glutaryl-coenzyme A reductase
IDL: intermediate-density lipoprotein
LDL: low-density lipoprotein
NMR: nuclear magnetic resonance
OR: odds ratio
PCSK9: proprotein convertase subtilisin-kexin type 9
SNP: single-nucleotide polymorphism
TG: triglycerides
VLDL: very-low-density lipoprotein
