# Correction: Lipoprotein signatures of cholesteryl ester transfer protein and HMG-CoA reductase inhibition

**DOI:** 10.1371/journal.pbio.3000694

**Published:** 2020-03-06

**Authors:** Johannes Kettunen, Michael V. Holmes, Elias Allara, Olga Anufrieva, Pauli Ohukainen, Clare Oliver-Williams, Qin Wang, Therese Tillin, Alun D. Hughes, Mika Kähönen, Terho Lehtimäki, Jorma Viikari, Olli T. Raitakari, Veikko Salomaa, Marjo-Riitta Järvelin, Markus Perola, George Davey Smith, Nish Chaturvedi, John Danesh, Emanuele Di Angelantonio, Adam S. Butterworth, Mika Ala-Korpela

As compared to the analysis presented in this work (using rs247616) [1], a similar Mendelian randomization analysis was presented in a recent paper by Blauw et al. for CETP (using rs247616, rs12720922 and rs1968905) [2]. The key findings regarding the genetic effects of CETP on cholesterol and triglycerides carried in various lipoprotein subclasses were very similar to the results presented in this article, despite the cohort used being more than ten-fold smaller (n = 5,672 in Blauw et al. vs. n = 65,427 in this article) [1,2]. In addition, Blauw et al. [2] present interesting observational data on the associations between circulating CETP concentrations and metabolic measures. The observational association profile was broadly similar to the genetic association profile only for the VLDL subclasses but striking differences were noted for IDL, LDL and HDL subclasses. Blauw et al. [2] hypothesised that the stronger affinity of CETP to VLDL, IDL and LDL subclass particles may hinder the HDL associations in the observational data. Regarding the most likely reason for CETP inhibition leading to a reduction in cardiovascular risk, Blauw et al. [2] arrived at the conclusion that it would likely be through a specific reduction of small VLDL rather than LDL, i.e., in full concordance with the findings presented here [1].
