# Supplementary material for: Lipoprotein signatures of cholesteryl ester transfer protein and HMG-CoA reductase inhibition
Source: PLoS Biol. 2019 Dec 20;17(12):e3000572. doi: 10.1371/journal.pbio.3000572 (PMC6944381; doi:10.1371/journal.pbio.3000572)
Supplement: S1 Text — Overall description of the individual cohorts. (PDF) [file pbio.3000572.s001.pdf]

Study descriptions

FINRISK & DILGOM

FINRISK surveys are cross-sectional, population-based studies conducted every five years since 1972 to monitor the risk of chronic diseases. For each survey, a representative random sample was selected from 24- to 74-year-old inhabitants of different regions in Finland. The survey included a questionnaire and a clinical examination, at which a blood sample was drawn, with linkage to national registers of cardiovascular and other health outcomes. The study protocol has been described elsewhere [1]. The current study included eligible individuals from FINRISK surveys conducted in 1997 (n = 6,942) and 2007 (n = 4,124). The FINRISK97 serum samples were from the clinical visit and individuals were requested to fast at least 4 hours. The DILGOM samples are from the 8-hour fasting subset of DIetary, Lifestyle, and Genetic determinants of Obesity and Metabolic syndrome (DILGOM) part of the 2007 survey [2]. The FINRISK samples were genotyped in various batches and arrays: PredictCVD (genotyped by HumanOmniExpress-12v1H), SANGER1 (genotyped by HumanCoreExome-12-v1-0-D), PSYCHO (genotyped by PsychArrayB), MRPRED (genotyped by HumanCoreExome-24v1-0A), SUMMIT (HumanOmniExpress-12v1H), MIGEN (genotyped by Affymetrix 6.0) and COROGENE (genotyped by Human 610-Quadv1H). Batches were imputed separately and later combined for analysis using batch as a covariate. In the genetic analyses individuals receiving lipid lowering medication (n = 269 in FINRISK and n = 761 in DILGOM), pregnant women (n = 75 in FINRISK and n = 22 in DILGOM) and those who had a high proportion (>30%) of values missing across the lipid traits (n = 1 in FINRISK and n = 1 in DILGOM) were excluded. In the observational analyses also patients with CHD at baseline were excluded (n = 113 in FINRISK and n = 22 in DILGOM).

Northern Finland Birth Cohorts (NFBCs)

The Northern Finland Birth Cohort studies are two longitudinal birth cohorts established to study factors affecting preterm birth and consequent morbidity in the two northernmost provinces of Finland, Oulu and Lapland. The NFBC66 includes 12,058 live births (12,231 children) covering 96% of all eligible births in this region during January-December 1966 [3]. The current study included eligible individuals from NFBC66 at age 31 years totaling up to n = 4,702 individuals. Two decades later, a second cohort of 9,432 births (9,479 children) was obtained (NFBC86) which covered 99% of all the deliveries taking place in the target regions during July 1985-June 1986 [4]. In both cohorts, mothers and children have been followed-up

since mothers enrolled at their first antenatal clinic visit (10-16th week). For NFBC86, data collection in 2001–2002 included clinical examination and serum sampling at age 15–16 for 6,621 adolescents; attendees in the 16-year field study (71% of invited participants) were representative of the original cohort. The current study included eligible individuals from NFBC86 totaling up to $n = 3{,}726$ individuals. In the genetic analyses individuals receiving lipid lowering medication ($n = 218$) and pregnant women ($n = 192$) were excluded in NFBC66. There were no individuals receiving lipid lowering medication or pregnant women in NFBC86. In both cohorts there were no individuals to exclude on the basis of a high proportion (>30%) of values missing across the lipid traits. Serum samples were drawn after overnight fasting.

Cardiovascular Risk in Young Finns Study (YFS)

The Cardiovascular Risk in Young Finns Study (YFS) is a population based prospective cohort study. It was conducted at five medical schools in Finland (Turku, Helsinki, Kuopio, Tampere and Oulu), with the aim of studying the levels of cardiovascular risk factors in children and adolescents in different parts of the country. The serum samples for this metabolomics study were collected from 2007 follow up. The study and data collection protocols have been described in detail by Raitakari *et al* [5]. The current study included eligible individuals from YFS totaling up to $n = 1{,}948$ individuals. In the genetic analyses individuals receiving lipid lowering medication ($n = 43$) and pregnant women ($n = 33$) were excluded. There were no individuals to exclude on the basis of a high proportion (>30%) of values missing across the lipid traits. Serum samples were drawn after overnight fasting.

INTERVAL

The INTERVAL Study is a prospective cohort study of approximately 50,000 participants nested within a pragmatic randomized trial of blood donors [6]. This study included 40,958 eligible individuals. Between 2012 and 2014, blood donors 18 years and older were consented and recruited from 25 NHSBT (National Health Service Blood and Transplant) static donor centers across England. Participants are predominantly healthy individuals since people with major disease (myocardial infarction, stroke, cancer etc) are ineligible for donation, as are those who report being unwell or having had recent illness or infection. Participants completed online questionnaires containing basic lifestyle and health-related information, including self-reported height and weight, ethnicity, current smoking status, alcohol consumption, doctor-diagnosed anemia, use of medications (hormone replacement therapy,

iron supplements) and menopausal status. A non-fasting research blood sample was taken before donation at the enrolment visit. Thirty-eight participants were removed from analysis due to a proportion of missing data > 30% across lipid traits. Because INTERVAL is primarily a trial of blood donors, pregnant women were excluded at the design stage as they are ineligible to donate blood, and information on lipid-lowering therapy was not collected during the baseline survey. Given the general good health of blood donors, this lack of information is unlikely to have substantially affected genetic estimates. At two years from baseline, 5% of INTERVAL participants (1,441 out of 28,396) were taking lipid-lowering medications. Although this information (which was not available when the genetic analyses for this study were performed) was collected at a different time point to when the blood samples were taken for the present study, retaining the small proportion of participants undergoing lipid-lowering therapy is unlikely to have affected the results substantially. This is further supported by the very close replications of genetic estimates from the other cohorts included in this study.

SABRE

The SABRE (Southall and Brent Revisited) study examined 4,976 individuals in a tri-ethnic community-based cohort from North and West London. In the observational analyses individuals receiving lipid lowering medication (n = 15) and those who had a high proportion (>30%) of values missing across the lipid traits (n = 319) were excluded. Also patients with CHD at baseline were excluded (n = 264). Information on pregnant women was not available. All participants gave written informed consent. Details of the cohort have been published [7,8]. Briefly, participants aged 40 to 69 at baseline (1988 through 1991) were selected randomly from 5-year age- and sex-stratified primary care physician lists. Participants included Europeans (48%), Indian Asians (47%) and a small fraction of African Caribbeans (5%). All statistical associations in the SABRE study were adjusted for ethnicity. Serum samples were drawn after overnight fasting.

References

1. Borodulin K, Vartiainen E, Peltonen M, Jousilahti P, Juolevi A, Laatikainen T, et al. Forty-year trends in cardiovascular risk factors in Finland. Eur J Public Health. 2015;25:539-46.
2. Konttinen H, Mannisto S, Sarlio-Lahteenkorva S, Silventoinen K, Haukkala A. Emotional eating, depressive symptoms and self-reported food consumption. A population-based study. Appetite. 2010;54:473–9.
3. Järvelin MR, Sovio U, King V, Lauren L, Xu B, McCarthy MI, et al. Early life factors and blood pressure at age 31 years in the 1966 Northern Finland birth cohort. Hypertension. 2004;44:838–46.
4. Järvelin MR, Hartikainen-Sorri AL, Rantakallio P. Labour induction policy in hospitals of different levels of specialisation. Br J Obs Gynaecol. 1993;100:310–5.
5. Raitakari OT, Juonala M, Rönnemaa T, Keltikangas-Järvinen L, Räsänen L. Cohort profile: The cardiovascular risk in young Finns study. Int J Epidemiol. 2008;37:1220–6.
6. Di Angelantonio E, Thompson SG, Kaptoge S, Moore C, Walker M, Armitage J, et al. Efficiency and safety of varying the frequency of whole blood donation (INTERVAL): A randomised trial of 45 000 donors. Lancet. 2017;390:2360-71.
7. Tillin T, Hughes AD, Mayet J, Whincup P, Sattar N, Forouhi NG, et al. The relationship between metabolic risk factors and incident cardiovascular disease in Europeans, South Asians, and African Caribbeans: SABRE (Southall and Brent Revisited) - A prospective population-based study. J Am Coll Cardiol. 2013;61:1777–86.
8. Tillin T, Forouhi NG, McKeigue PM, Chaturvedi N, Chaturvedi N, Beauchamp N, et al. Southall And Brent REvisited: Cohort profile of SABRE, a UK population-based comparison of cardiovascular disease and diabetes in people of European, Indian Asian and African Caribbean origins. Int J Epidemiol. 2012;41:33–42.
