# Supplementary material for: Lipoprotein signatures of cholesteryl ester transfer protein and HMG-CoA reductase inhibition
Source: PLoS Biol. 2019 Dec 20;17(12):e3000572. doi: 10.1371/journal.pbio.3000572 (PMC6944381; doi:10.1371/journal.pbio.3000572)
Supplement: S1 Table — Clinical characteristics for the 7 cohorts included in the study. (PDF) [file pbio.3000572.s002.pdf]

**Clinical characteristics**	**FINRISK97**	**DILGOM**	**YFS**	**NFBC66**	**NFBC86**	**INTERVAL**	**SABRE**
Number of participants	6,942	4,124	1,948	4,702	3,726	40,958	4,976
Age (years ± s.d.)	47.5 ± 13.2	52.1 ± 13.6	37.7 ± 5.0	31.0 ± 0.3	16 ± 0.4	43.5 ± 14.3	52.4 ± 7.0
Female (%)	50	53	53	54	48	51	22
BMI ± s.d.	26.7 ± 4.5	26.5 ± 4.6	26.0 ± 4.7	24.6 ± 4.2	21.3 ± 3.6	26.4 ± 4.6	26.4 ± 4.0
Total cholesterol (mmol/l)	5.0 ± 1.0	4.3 ± 0.9	5.0 ± 1.0	5.3 ± 1.2	4.4 ± 0.9	4.2 ± 1.0	5.9 ± 1.1
HDL cholesterol (mmol/l)	1.6 ± 0.3	1.5 ± 0.4	1.6 ± 0.3	1.7 ± 0.4	1.5 ± 0.3	1.4 ± 0.4	1.4 ± 0.4
Triglycerides (mmol/l)	0.9 ± 0.6	1.1 ± 0.6	1.3 ± 0.7	1.2 ± 0.7	0.9 ± 0.5	1.2 ± 0.5	1.7 ± 1.3
