# Supplementary material for: Lipoprotein signatures of cholesteryl ester transfer protein and HMG-CoA reductase inhibition
Source: PLoS Biol. 2019 Dec 20;17(12):e3000572. doi: 10.1371/journal.pbio.3000572 (PMC6944381; doi:10.1371/journal.pbio.3000572)
Supplement: S2 Table — Details of the genotyping for the 6 cohorts including genetic data in this study. (PDF) [file pbio.3000572.s003.pdf]

Study	Short name	FINRISK97	DILGOM	YFS	NFBC66	NFBC86	INTERVAL
	**Full name**	FINRISK97	DIetary, Lifestyle, and Genetic determinants of Obesity and Metabolic syndrome	The Cardiovascular Risk in Young Finns Study	Northern Finland Birth Cohort Study 1966	Northern Finland Birth Cohort Study 1986	INTERVAL
**Study design**		Population-based cohort	Population-based cohort	Follow up study in children	Birth cohort	Birth cohort	Follow up study in blood donors
**Ethnicity**		Finnish	Finnish	Finnish	Finnish	Finnish	European ancestries
**Sample QC**	**Call rate**	≥ 95%	≥ 95%	≥ 95%	≥ 95%	≥ 95%	≥ 95%
	**Other exclusions**	IBD > 0.1, sex mismatches, duplicates, heterozygosity outliers by eye from distribution	IBD > 0.1, sex mismatches, duplicates, heterozygosity outliers by eye from distribution	IBD > 0.1, sex mismatches, duplicates, heterozygosity outliers by eye from distribution	IBD > 0.2, sex mismatches, duplicates, heterozygosity outliers by eye from distribution	IBD pairwise sharing>0.20; sex mismatch, duplicates, heterozygosity outliers by eye from distribution	Sex mismatches, duplicates, contaminated samples, heterozygosity outliers (>3SD from the mean), non-European samples by PCA
**Genotyping**	**Array used**	Several platforms †	Several platforms †	llumina 670k	Illumina 370k	Illumina HumanOmniExpressExome	Affymetrix UK Biobank Axiom
	**Genotype calling algorithm**	Illuminus	Illuminus	Illuminus	Illuminus	Genomestudio	Affymetrix Axiom GT1
	**MAF; p for HWE; Info (rs247617)**	0.28; 0.06; 0.99	0.28; 0.06; 0.99	0.27; 0.96; 1	0.28; 0.50; 1	0.27; 0.34; 1	0.32; 0.41;1.0
	**MAF; p for HWE; Info (rs12916)**	0.46; 0.56; 0.99	0.46; 0.56; 0.99	0.45; 0.33; 1	0.44; 0.91; 1	0.43; 0.3; 1	0.40; 0.68; 0.99
	**Imputation software and panel**	IMPUTE2, 1000 genomes phase 1v3	IMPUTE2, 1000 genomes phase 1v3	HRC imputation pipeline	HRC imputation pipeline	HRC imputation pipeline	Sanger imputation server (SHAPEIT3, PBWT), 1000G Phase3 & UK10K
	**Analysis software**	SNPTEST 2.5.1	SNPTEST 2.5.1	SNPTEST 2.5.1	SNPTEST 2.5.1	SNPTEST 2.5.1	BOLT-LMM 2.2

† Please see cohort descriptions for different genotyping batches.
