# Supplementary material for: Lipoprotein signatures of cholesteryl ester transfer protein and HMG-CoA reductase inhibition
Source: PLoS Biol. 2019 Dec 20;17(12):e3000572. doi: 10.1371/journal.pbio.3000572 (PMC6944381; doi:10.1371/journal.pbio.3000572)
Supplement: S3 Table — Abbreviations and the description of the lipid and lipoprotein subclass measures. (PDF) [file pbio.3000572.s004.pdf]

Trait abbreviation	Trait description
	**LIPOPROTEIN SUBCLASSES**
**XXL-VLDL**	Chylomicrons and extremely large very-low-density lipoprotein (VLDL) particles (average particle diameter ≥75 nm)
**XL-VLDL**	Very large VLDL particles (64 nm)
**L-VLDL**	Large VLDL particles (53.6 nm)
**M-VLDL**	Medium VLDL particles (44.5 nm)
**S-VLDL**	Small VLDL particles (36.8 nm)
**XS-VLDL**	Very small VLDL particles (31.3 nm)
**IDL**	Intermediate-density lipoprotein particles (28.6 nm)
**L-LDL**	Large low-density lipoprotein (LDL) particles (25.5 nm)
**M-LDL**	Medium (LDL) particles (23.0 nm)
**S-LDL**	Small (LDL) particles (18.7 nm)
**XL-HDL**	Very large high-density lipoprotein (HDL) particles (14.3 nm)
**L-HDL**	Large (HDL) particles (12.1 nm)
**M-HDL**	Medium (HDL) particles (10.9 nm)
**S-HDL**	Small (HDL) particles (8.7 nm)
	**CONCENTRATIONS OF CIRCULATING LIPIDS**
**P**	Particle concentration (mol/l)
**L**	Total lipids (mmol/l)
**PL**	Phospholipids (mmol/l)
**C**	Total cholesterol (mmol/l)
**CE**	Cholesterol esters (mmol/l)
**FC**	Free cholesterol (mmol/l)
**TG**	Triglycerides (mmol/l)
	**LIPOPROTEIN COMPOSITION MEASURES**
**PL%**	Phospholipids to total lipids ratio (%)
**C%**	Total cholesterol to total lipids ratio (%)
**CE%**	Cholesterol esters to total lipids ratio (%)
**FC%**	Free cholesterol to total lipids ratio (%)
**TG%**	Triglycerides to total lipids ratio (%)
	**AVERAGE PARTICLE DIAMETER**
**VLDL-D**	Mean diameter for VLDL particles (nm)

**LDL-D**	Mean diameter for LDL particles (nm)
**HDL-D**	Mean diameter for HDL particles (nm)
	**TOTAL CIRCULATING LIPID CONCENTRATIONS**
**Serum-C**	Serum total cholesterol (mmol/l)
**VLDL-C**	Total cholesterol in VLDL particles (mmol/l)
**Remnant-C**	Remnant cholesterol (non-HDL, non-LDL cholesterol) (mmol/l)
**LDL-C**	Total cholesterol in LDL particles (mmol/l)
**HDL-C**	Total cholesterol in HDL particles (mmol/l)
**HDL2-C**	Total cholesterol in HDL2 particles (mmol/l)
**HDL3-C**	Total cholesterol in HDL3 particles (mmol/l)
**EstC**	Esterified cholesterol (mmol/l)
**FreeC**	Free cholesterol (mmol/l)
**Serum-TG**	Serum total triglycerides (mmol/l)
**VLDL-TG**	Triglycerides in VLDL particles (mmol/l)
**LDL-TG**	Triglycerides in LDL particles (mmol/l)
**HDL-TG**	Triglycerides in HDL particles (mmol/l)
**TotPG**	Total phosphoglycerides (mmol/l)
**TG/PG**	Ratio of triglycerides to phosphoglycerides
**PC**	Phosphatidylcholine and other cholines (mmol/l)
**SM**	Sphingomyelins (mmol/l)
**TotCho**	Total cholines (mmol/l)
	**APOLIPOPROTEIN CONCENTRATIONS**
**ApoA-I**	Apolipoprotein A-I (g/l)
**ApoB**	Apolipoprotein B (g/l)
