# Supplementary figures and images for: Lipoprotein signatures of cholesteryl ester transfer protein and HMG-CoA reductase inhibition
Source: PLoS Biol. 2019 Dec 20;17(12):e3000572. doi: 10.1371/journal.pbio.3000572 (PMC6944381; doi:10.1371/journal.pbio.3000572)

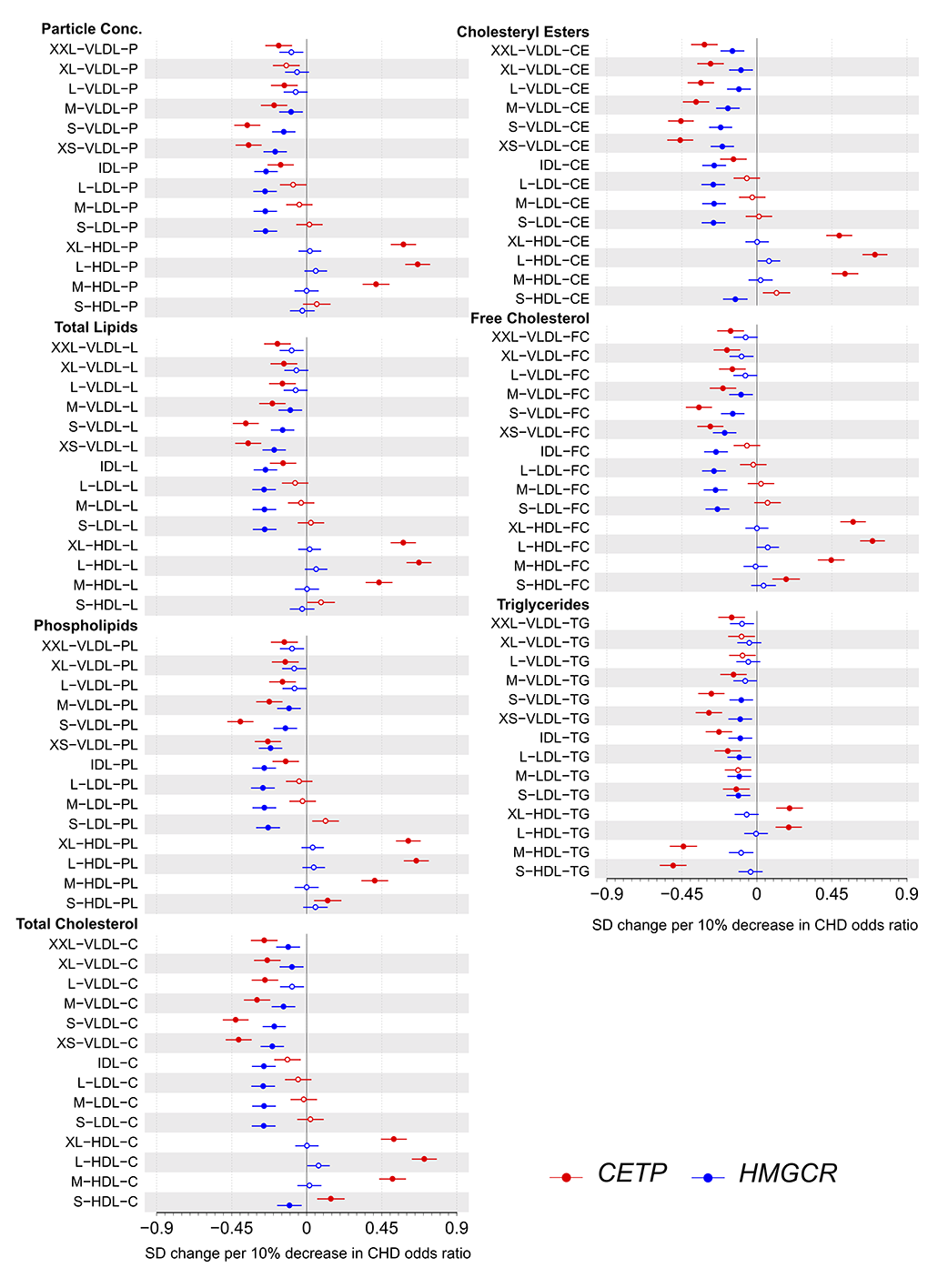

Supplement: S1 Fig — Meta-analysis of genetic variants in CETP rs247617 (red) and HMGCR rs12916 (blue) for all lipoprotein concentration measures. Estimates are the standardized difference in lipoprotein trait, with per allele associations scaled to a 10% lower relative risk of CHD. The circles refer to the effect estimates and the horizontal bars to the 95% CIs. Closed circles represent associations at P < 0.002, and open circles show associations that are nonsignificant at this threshold. The lipoprotein subclasses are defined by particle size: potential chylomicrons and the largest VLDL particles (XXL-VLDL; average particle diameter ≥75 nm); 5 different VLDL subclasses, i.e., very large (average particle diameter 64.0 nm), large (53.6 nm), medium (44.5 nm), small (36.8 nm), and very small (31.3 nm); IDL (28.6 nm); and 3 LDL subclasses, i.e., large (25.5 nm), medium (23.0 nm), and small (18.7 nm). Underlying data can be found in S2 Data. CHD, coronary heart disease; IDL, intermediate-density lipoprotein; LDL, low-density lipoprotein; VLDL, very-low-density lipoprotein. (TIF) [file pbio.3000572.s005.tif]

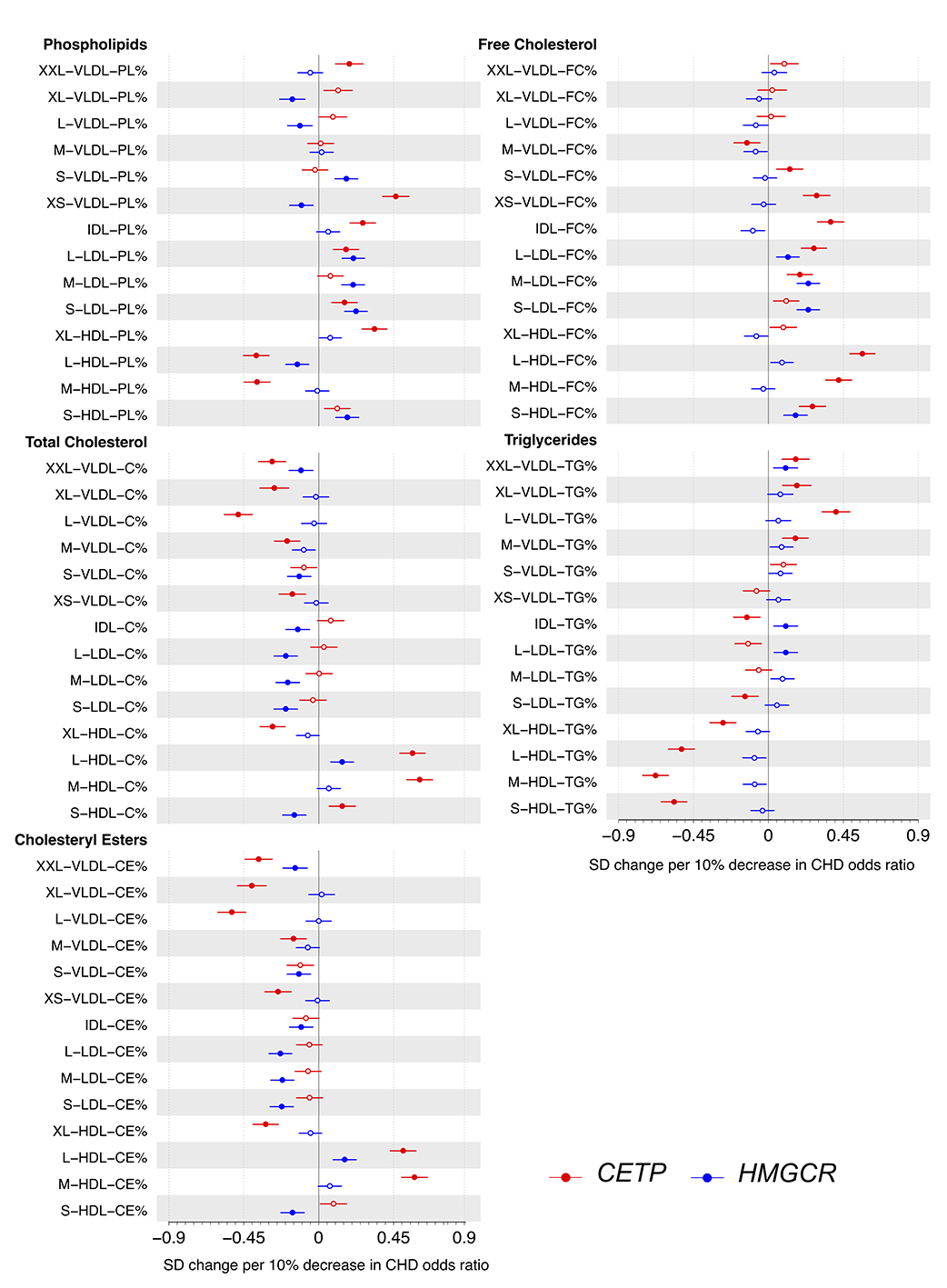

Supplement: S2 Fig — Meta-analysis of genetic variants in CETP rs247617 (red) and HMGCR rs12916 (blue) for all lipoprotein composition measures. Estimates are the standardized difference in lipoprotein trait, with per allele associations scaled to a 10% lower relative risk of CHD. The circles refer to the effect estimates and the horizontal bars to the 95% CIs. Closed circles represent associations at P < 0.002, and open circles show associations that are nonsignificant at this threshold. The lipoprotein subclasses are defined by particle size: potential chylomicrons and the largest VLDL particles (XXL-VLDL; average particle diameter ≥75 nm); 5 different VLDL subclasses, i.e., very large (average particle diameter 64.0 nm), large (53.6 nm), medium (44.5 nm), small (36.8 nm), and very small (31.3 nm); IDL (28.6 nm); and 3 LDL subclasses, i.e., large (25.5 nm), medium (23.0 nm), and small (18.7 nm). Underlying data can be found in S2 Data. CHD, coronary heart disease; IDL, intermediate-density lipoprotein; LDL, low-density lipoprotein; VLDL, very-low-density lipoprotein. (TIF) [file pbio.3000572.s006.tif]

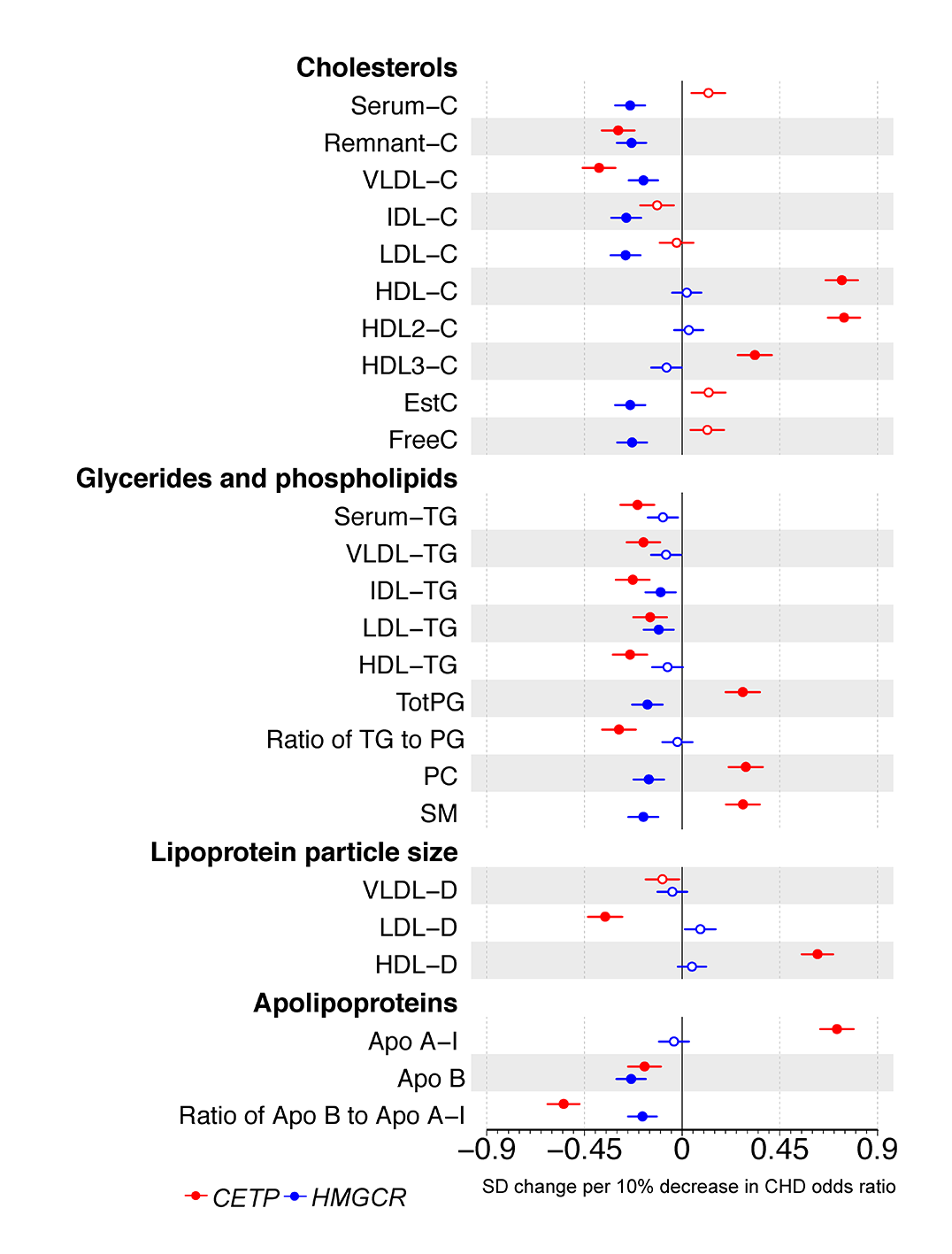

Supplement: S3 Fig — Meta-analysis of genetic variants in CETP rs247617 (red) and HMGCR rs12916 (blue) for all summary lipid measures. Estimates are the standardized difference in lipoprotein trait, with per allele associations scaled to a 10% lower relative risk of CHD. The circles refer to the effect estimates and the horizontal bars to the 95% CIs. Closed circles represent associations at P < 0.002, and open circles show associations that are nonsignificant at this threshold. The lipoprotein subclasses are defined by particle size: potential chylomicrons and the largest VLDL particles (XXL-VLDL; average particle diameter ≥75 nm); 5 different VLDL subclasses, i.e., very large (average particle diameter 64.0 nm), large (53.6 nm), medium (44.5 nm), small (36.8 nm), and very small (31.3 nm); IDL (28.6 nm); and 3 LDL subclasses, i.e., large (25.5 nm), medium (23.0 nm), and small LDL (18.7 nm). Underlying data can be found in S2 Data. CHD, coronary heart disease; IDL, intermediate-density lipoprotein; LDL, low-density lipoprotein; VLDL, very-low-density lipoprotein. (TIF) [file pbio.3000572.s007.tif]

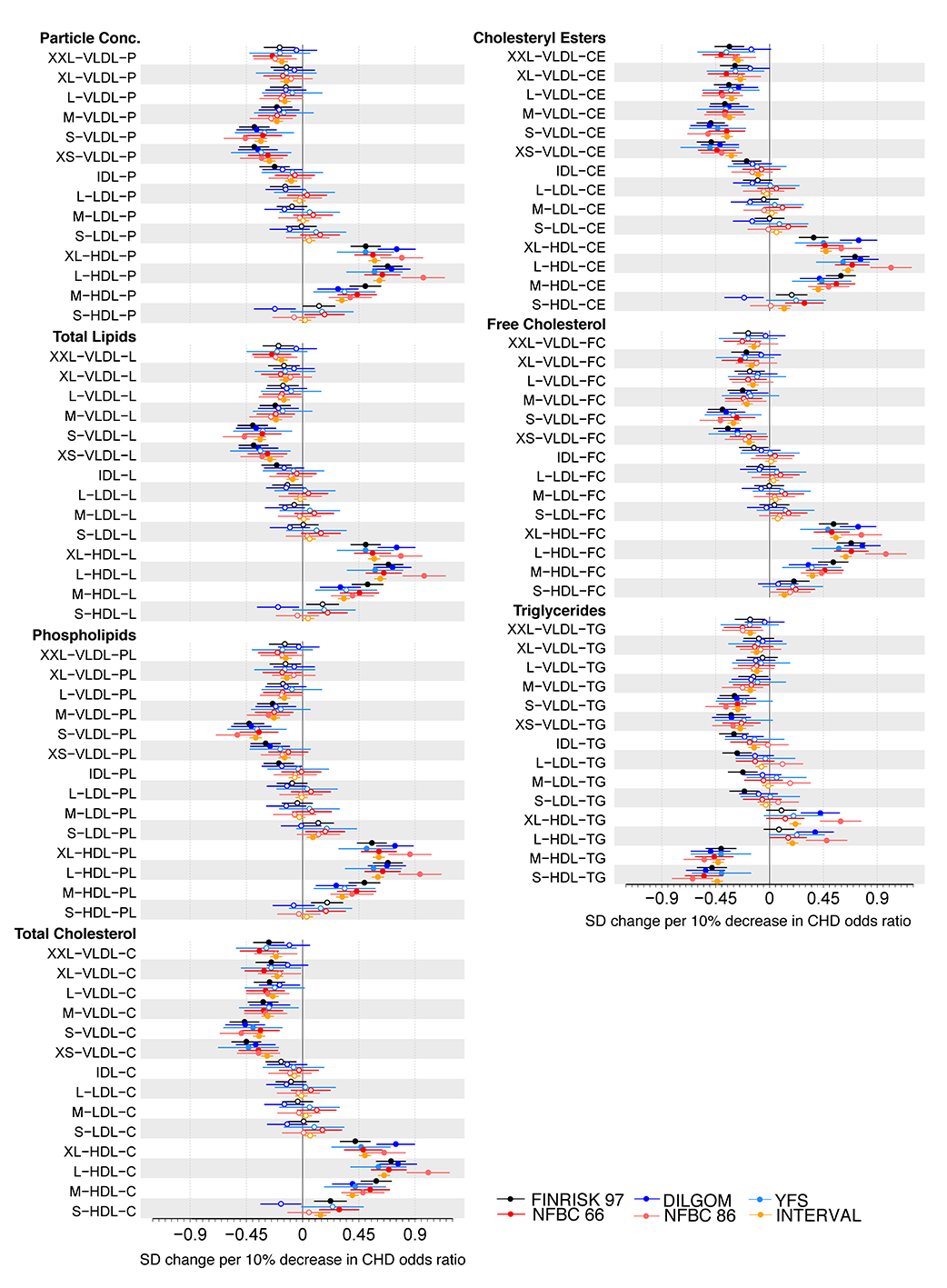

Supplement: S4 Fig — Estimates are the standardized difference in lipoprotein trait, with per allele associations scaled to a 10% lower relative risk of CHD. The circles refer to the effect estimates and the horizontal bars to the 95% CIs. Closed circles represent associations at P < 0.002, and open circles show associations that are nonsignificant at this threshold. The lipoprotein subclasses are defined by particle size: potential chylomicrons and the largest VLDL particles (XXL-VLDL; average particle diameter ≥75 nm); 5 different VLDL subclasses, i.e., very large (average particle diameter 64.0 nm), large (53.6 nm), medium (44.5 nm), small (36.8 nm), and very small (31.3 nm); IDL (28.6 nm); and 3 LDL subclasses, i.e., large (25.5 nm), medium (23.0 nm), and small (18.7 nm). Underlying data can be found in S2 Data. CHD, coronary heart disease; IDL, intermediate-density lipoprotein; LDL, low-density lipoprotein; VLDL, very-low-density lipoprotein. (TIF) [file pbio.3000572.s008.tif]

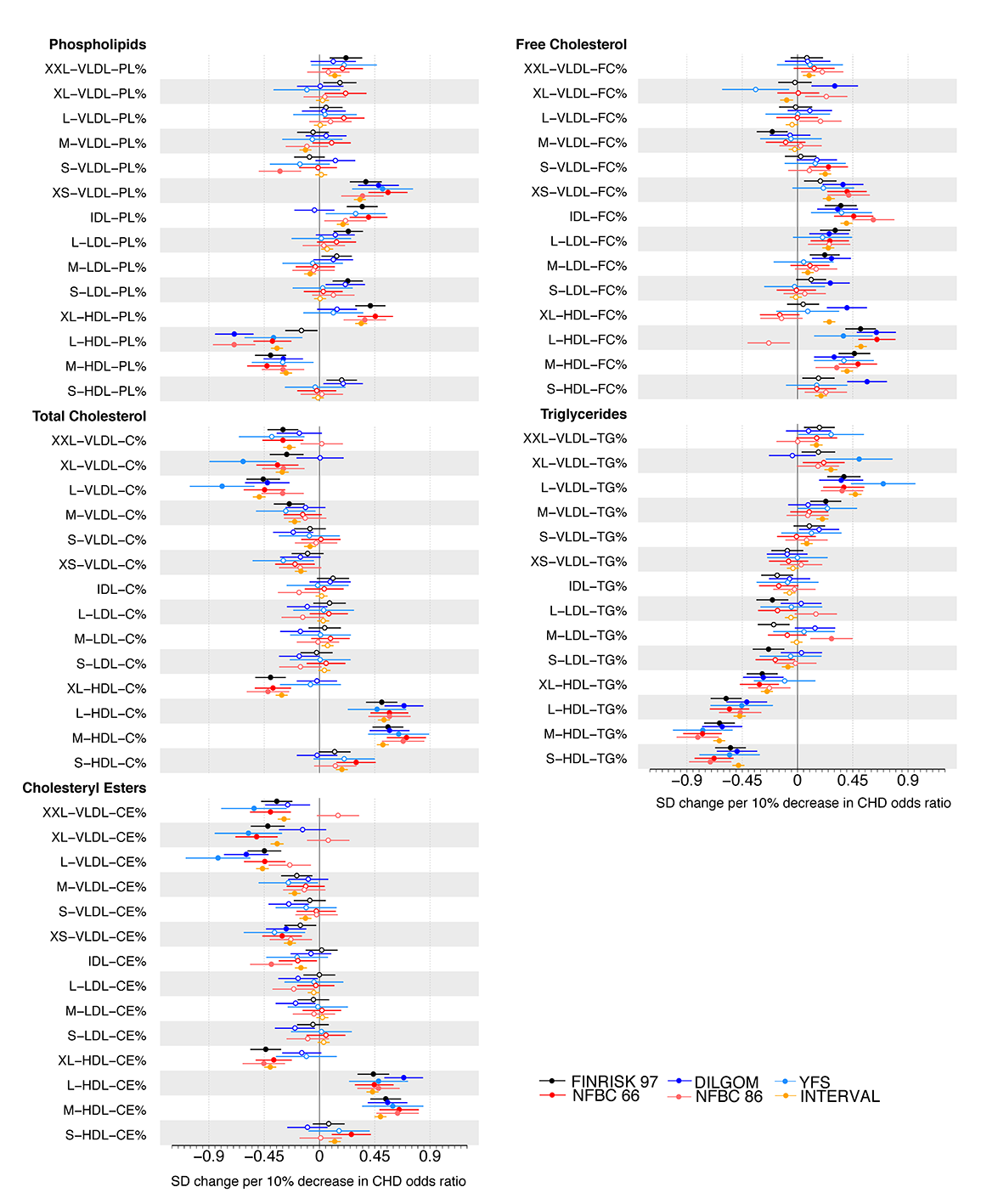

Supplement: S5 Fig — Estimates are the standardized difference in lipoprotein trait, with per allele associations scaled to a 10% lower relative risk of CHD. The circles refer to the effect estimates and the horizontal bars to the 95% CIs. Closed circles represent associations at P < 0.002, and open circles show associations that are nonsignificant at this threshold. The lipoprotein subclasses are defined by particle size: potential chylomicrons and the largest VLDL particles (XXL-VLDL; average particle diameter ≥75 nm); 5 different VLDL subclasses, i.e., very large (average particle diameter 64.0 nm), large (53.6 nm), medium (44.5 nm), small (36.8 nm), and very small (31.3 nm); IDL (28.6 nm); and 3 LDL subclasses, i.e., large (25.5 nm), medium (23.0 nm), and small (18.7 nm). Underlying data can be found in S2 Data. CHD, coronary heart disease; IDL, intermediate-density lipoprotein; LDL, low-density lipoprotein; VLDL, very-low-density lipoprotein. (TIF) [file pbio.3000572.s009.tif]

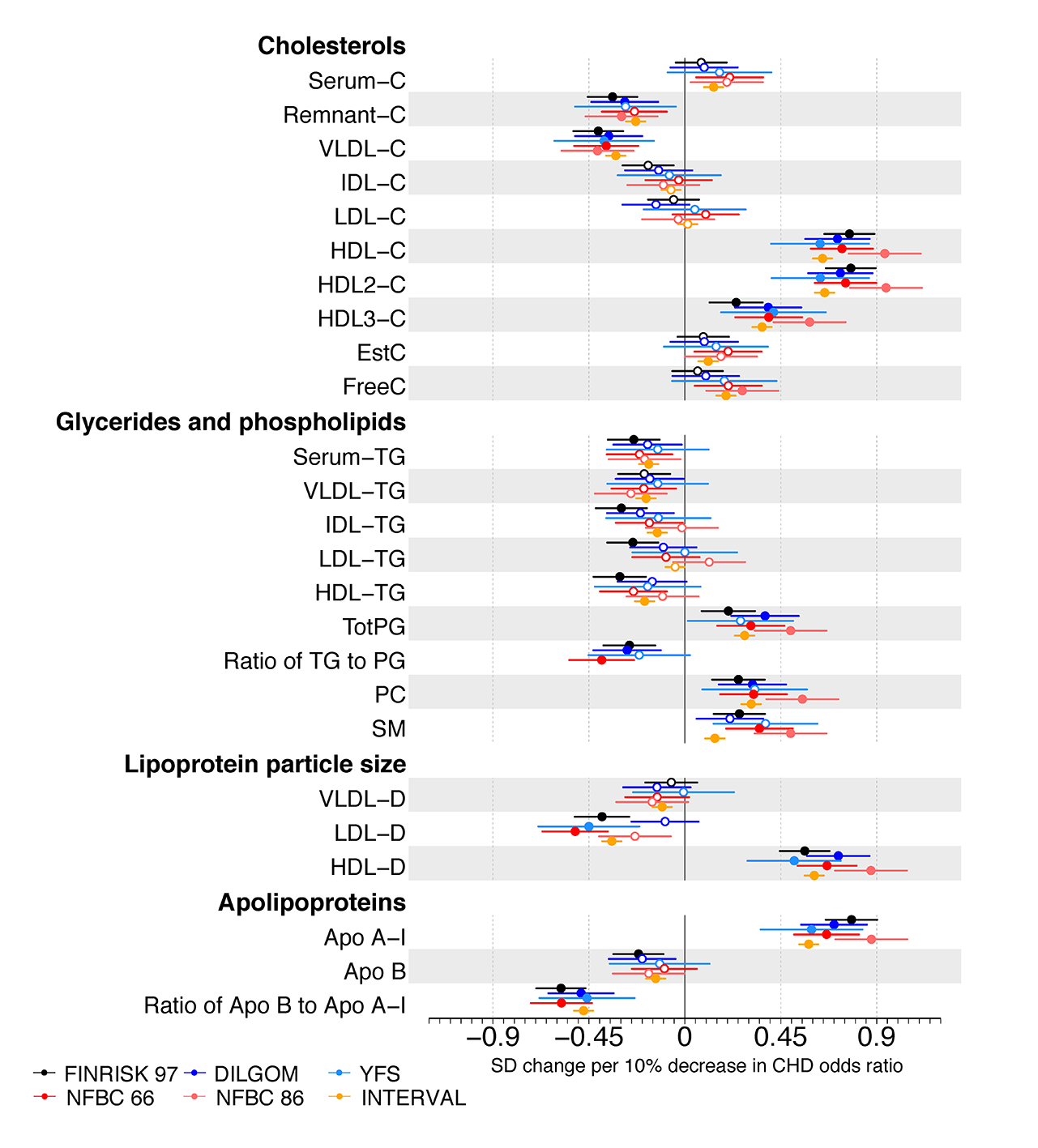

Supplement: S6 Fig — Estimates are the standardized difference in lipoprotein trait, with per allele associations scaled to a 10% lower relative risk of CHD. The circles refer to the effect estimates and the horizontal bars to the 95% CIs. Closed circles represent associations at P < 0.002, and open circles show associations that are nonsignificant at this threshold. The lipoprotein subclasses are defined by particle size: potential chylomicrons and the largest VLDL particles (XXL-VLDL; average particle diameter ≥75 nm); 5 different VLDL subclasses, i.e., very large (average particle diameter 64.0 nm), large (53.6 nm), medium (44.5 nm), small (36.8 nm), and very small (31.3 nm); IDL (28.6 nm); and 3 LDL subclasses, i.e., large (25.5 nm), medium (23.0 nm), and small (18.7 nm). Underlying data can be found in S2 Data. CHD, coronary heart disease; IDL, intermediate-density lipoprotein; LDL, low-density lipoprotein; VLDL, very-low-density lipoprotein. (TIF) [file pbio.3000572.s010.tif]

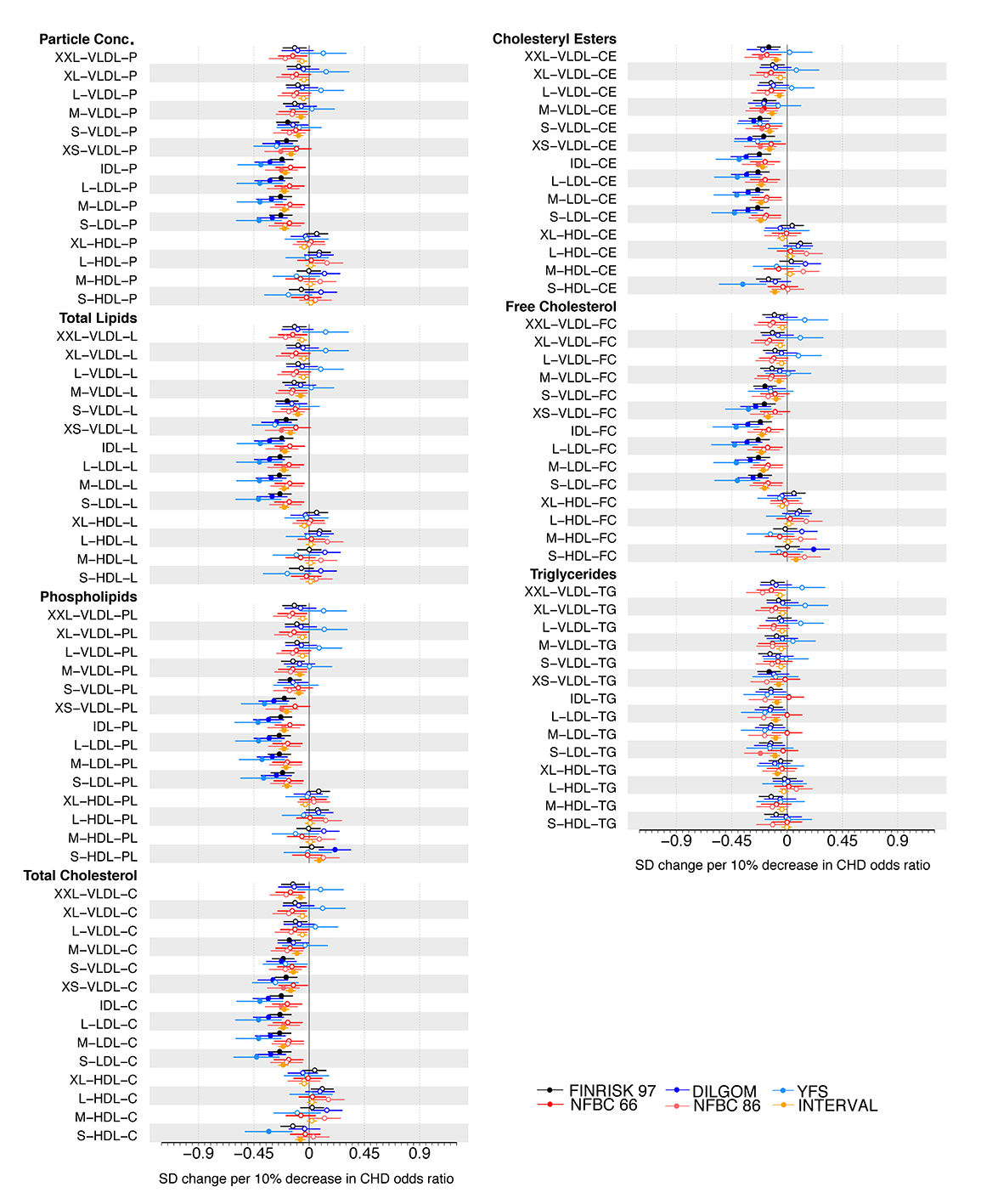

Supplement: S7 Fig — Estimates are the standardized difference in lipoprotein trait, with per-allele associations scaled to a 10% lower relative risk of CHD. The circles refer to the effect estimates and the horizontal bars to the 95% CIs. Closed circles represent associations at P < 0.002, and open circles show associations that are nonsignificant at this threshold. The lipoprotein subclasses are defined by particle size: potential chylomicrons and the largest VLDL particles (XXL-VLDL; average particle diameter ≥75 nm); 5 different VLDL subclasses, i.e., very large (average particle diameter 64.0 nm), large (53.6 nm), medium (44.5 nm), small (36.8 nm), and very small (31.3 nm); IDL (28.6 nm); and 3 LDL subclasses, i.e., large (25.5 nm), medium (23.0 nm), and small (18.7 nm). Underlying data can be found in S2 Data. CHD, coronary heart disease; IDL, intermediate-density lipoprotein; LDL, low-density lipoprotein; VLDL, very-low-density lipoprotein. (TIF) [file pbio.3000572.s011.tif]

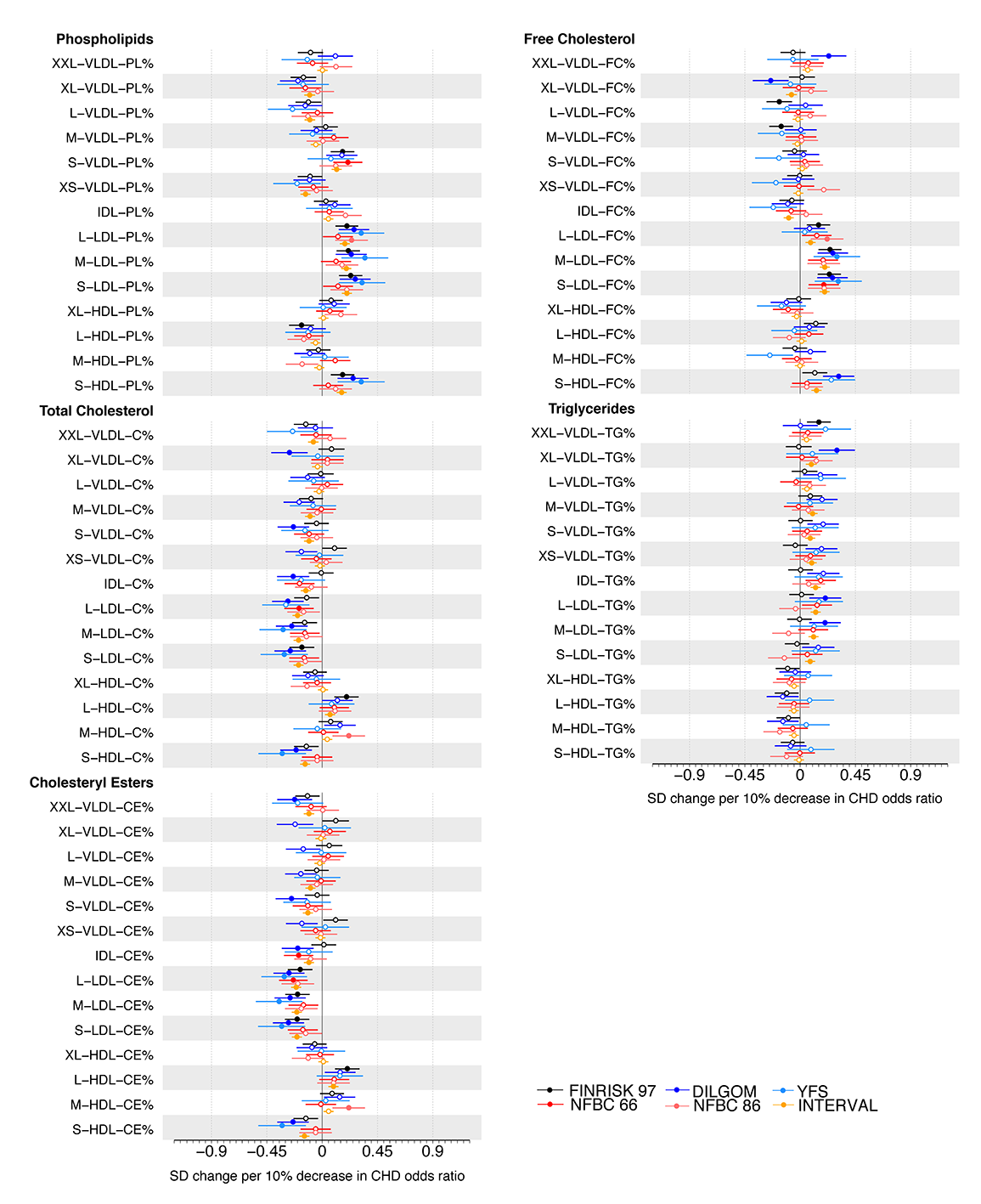

Supplement: S8 Fig — Estimates are the standardized difference in lipoprotein trait, with per allele associations scaled to a 10% lower relative risk of CHD. The circles refer to the effect estimates and the horizontal bars to the 95% CIs. Closed circles represent associations at P < 0.002, and open circles show associations that are nonsignificant at this threshold. The lipoprotein subclasses are defined by particle size: potential chylomicrons and the largest VLDL particles (XXL-VLDL; average particle diameter ≥75 nm); 5 different VLDL subclasses, i.e., very large (average particle diameter 64.0 nm), large (53.6 nm), medium (44.5 nm), small (36.8 nm), and very small (31.3 nm); IDL (28.6 nm); and 3 LDL subclasses, i.e., large (25.5 nm), medium (23.0 nm), and small (18.7 nm). Underlying data can be found in S2 Data. CHD, coronary heart disease; IDL, intermediate-density lipoprotein; LDL, low-density lipoprotein; VLDL, very-low-density lipoprotein. (TIF) [file pbio.3000572.s012.tif]

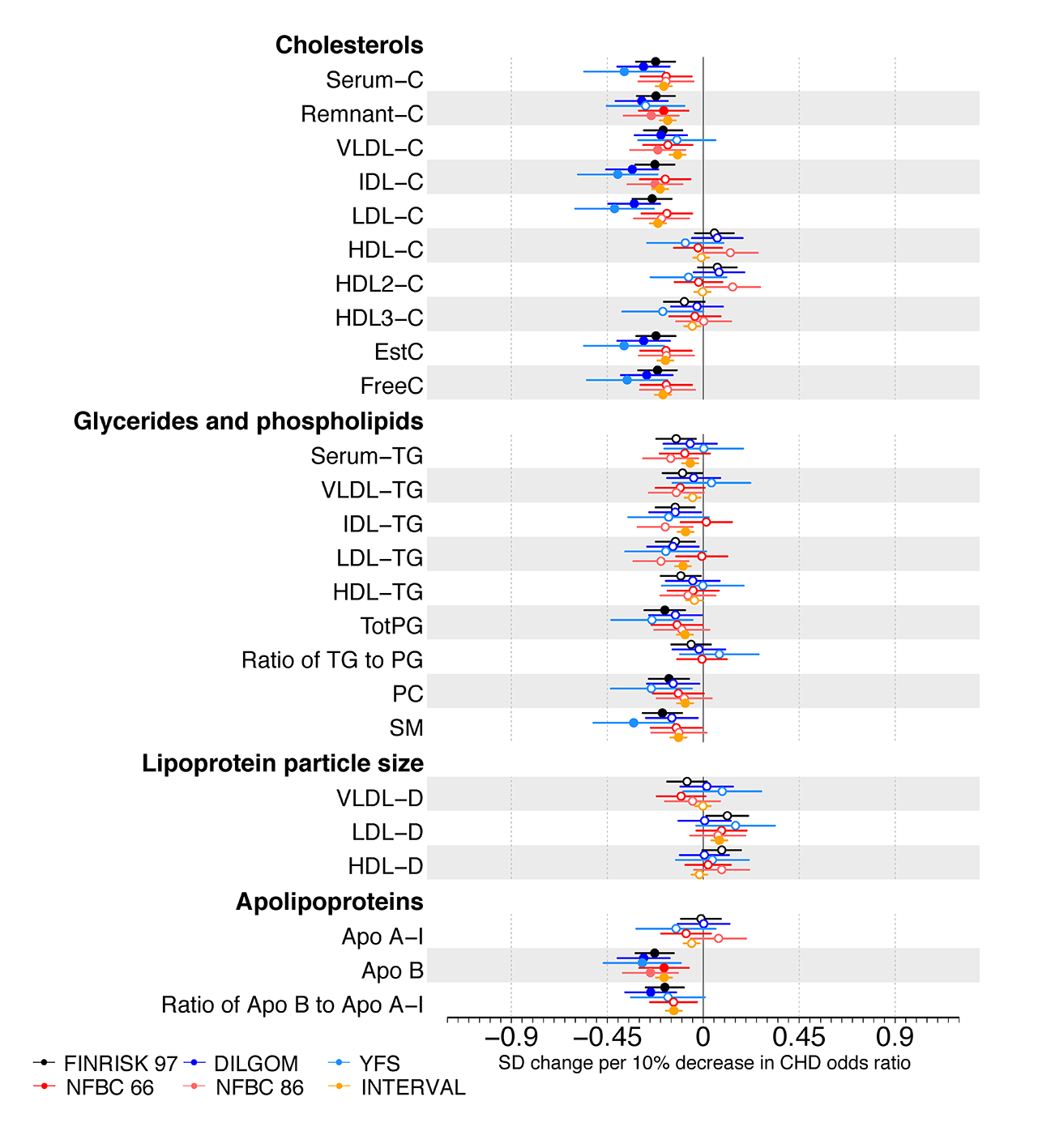

Supplement: S9 Fig — Estimates are the standardized difference in lipoprotein trait, with per allele associations scaled to a 10% lower relative risk of CHD. The circles refer to the effect estimates and the horizontal bars to the 95% CIs. Closed circles represent associations at P < 0.002, and open circles show associations that are nonsignificant at this threshold. The lipoprotein subclasses are defined by particle size: potential chylomicrons and the largest VLDL particles (XXL-VLDL; average particle diameter ≥75 nm); 5 different VLDL subclasses, i.e., very large (average particle diameter 64.0 nm), large (53.6 nm), medium (44.5 nm), small (36.8 nm), and very small (31.3 nm); IDL; 28.6 nm); and 3 LDL subclasses, i.e., large (25.5 nm), medium (23.0 nm), and small (18.7 nm). Underlying data can be found in S2 Data. CHD, coronary heart disease; IDL, intermediate-density lipoprotein; LDL, low-density lipoprotein; VLDL, very-low-density lipoprotein. (TIF) [file pbio.3000572.s013.tif]

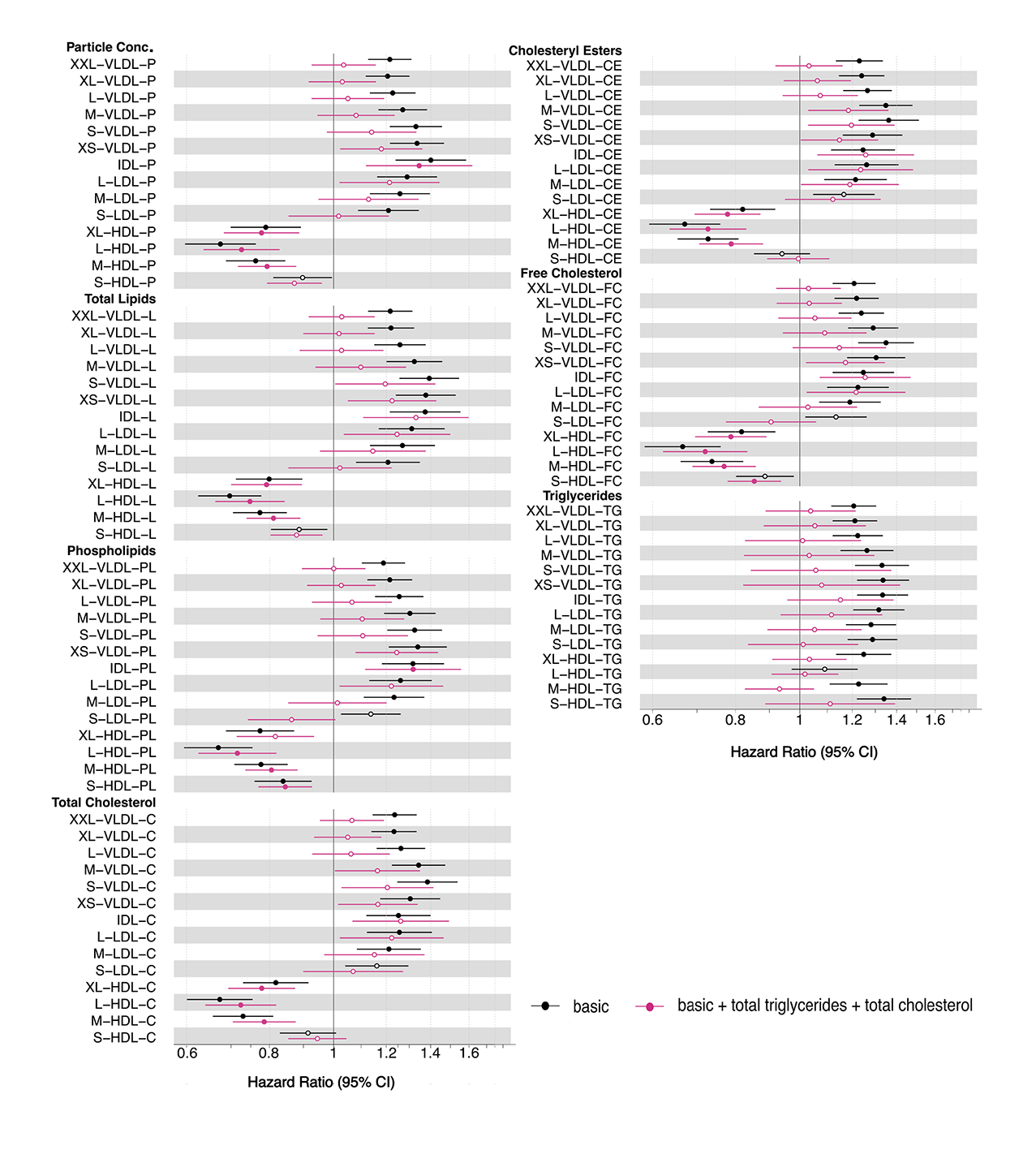

Supplement: S10 Fig — Estimates represent hazard ratios for incident CHD per SD lipoprotein concentration. Black color refers to adjusting for the traditional risk factors and pink color adjusting for the traditional risk factors and serum cholesterol and serum triglycerides. Traditional risk factors include age, sex, mean arterial pressure, smoking, diabetes mellitus, lipid medication, geographical region in FINRISK, and ethnicity in SABRE. Closed circles represent statistical significance of associations at P < 0.002 and open circles associations that are nonsignificant at this threshold. The horizontal bars refer to the 95% CIs. Underlying data can be found in S2 Data. CHD, coronary heart disease. (TIF) [file pbio.3000572.s014.tif]

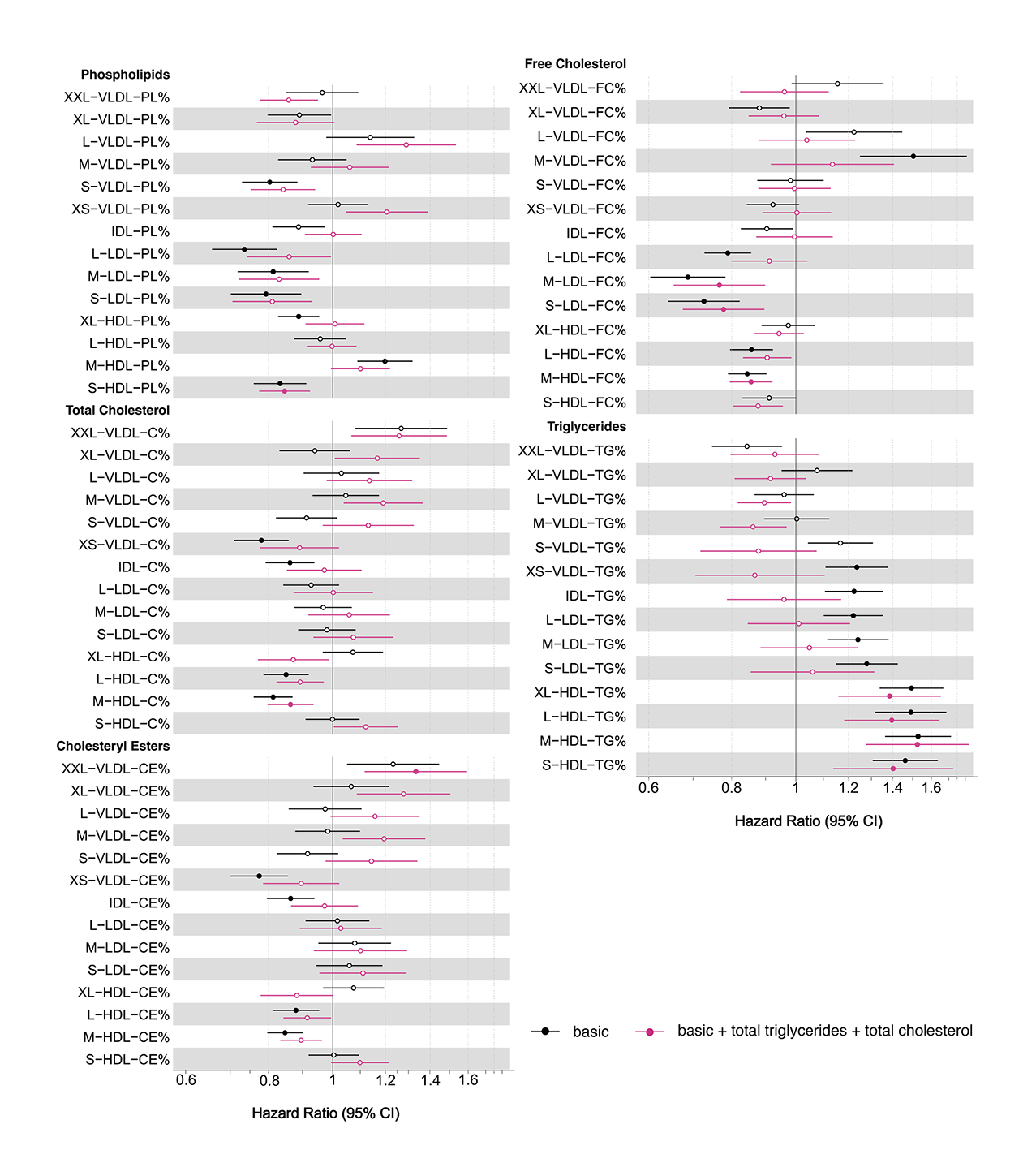

Supplement: S11 Fig — Estimates represent hazard ratios for incident CHD per SD lipoprotein composition measure. Black color refers to adjusting for the traditional risk factors and pink color adjusting for the traditional risk factors and serum cholesterol and serum triglycerides. Traditional risk factors include age, sex, mean arterial pressure, smoking, diabetes mellitus, lipid medication, geographical region in FINRISK, and ethnicity in SABRE. Closed circles represent statistical significance of associations at P < 0.002 and open circles associations that are nonsignificant at this threshold. The horizontal bars refer to the 95% CIs. Underlying data can be found in S2 Data. CHD, coronary heart disease. (TIF) [file pbio.3000572.s015.tif]

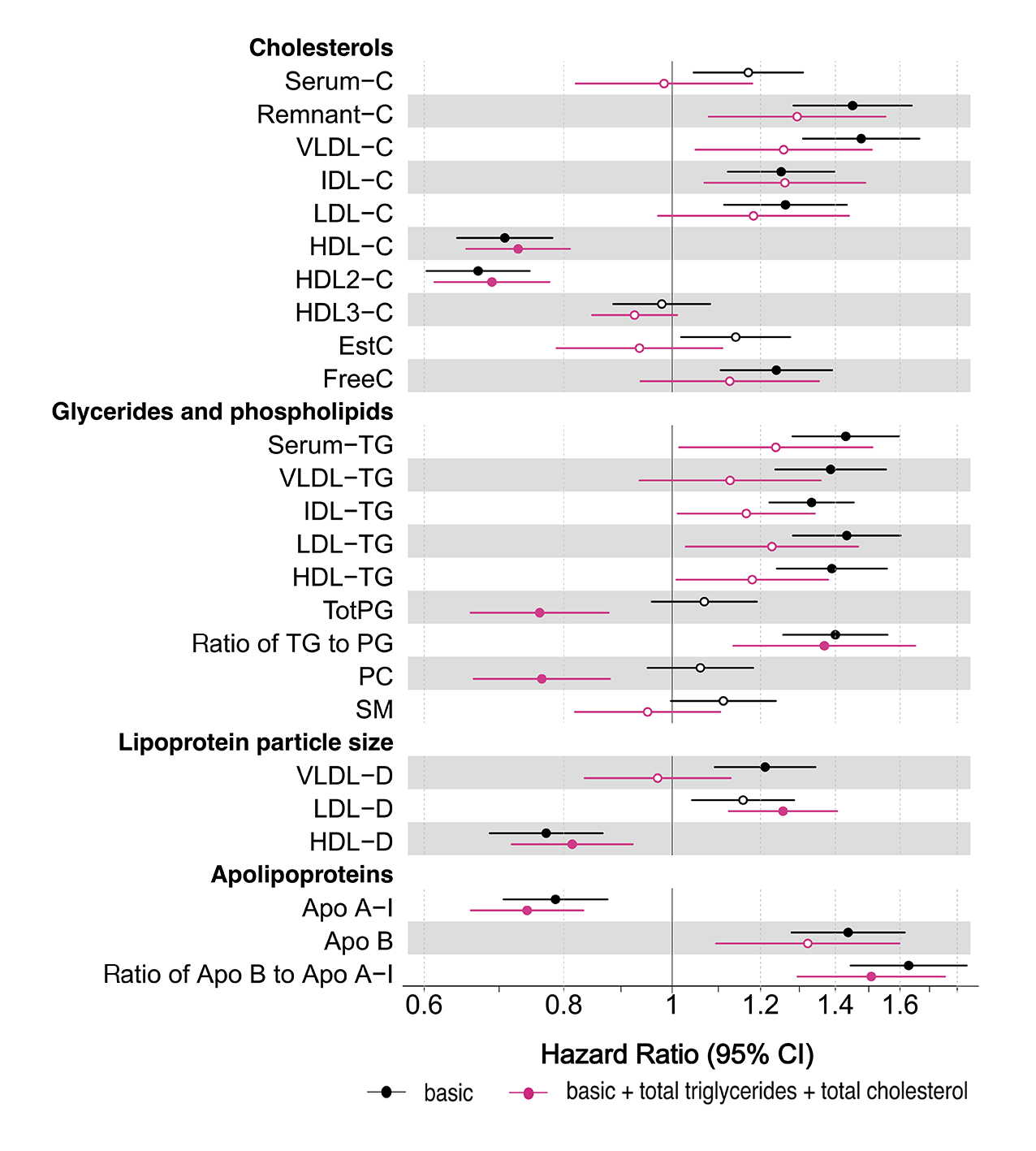

Supplement: S12 Fig — Estimates represent hazard ratios for incident CHD per SD lipoprotein measure. Black colo r refers to adjusting for the traditional risk factors and pink color adjusting for the traditional risk factors and serum cholesterol and serum triglycerides. Traditional risk factors include age, sex, mean arterial pressure, smoking, diabetes mellitus, lipid medication, geographical region in FINRISK, and ethnicity in SABRE. Closed circles represent statistical significance of associations at P < 0.002 and open circles associations that are nonsignificant at this threshold. The horizontal bars refer to the 95% CIs. Underlying data can be found in S2 Data. CHD, coronary heart disease. (TIF) [file pbio.3000572.s016.tif]

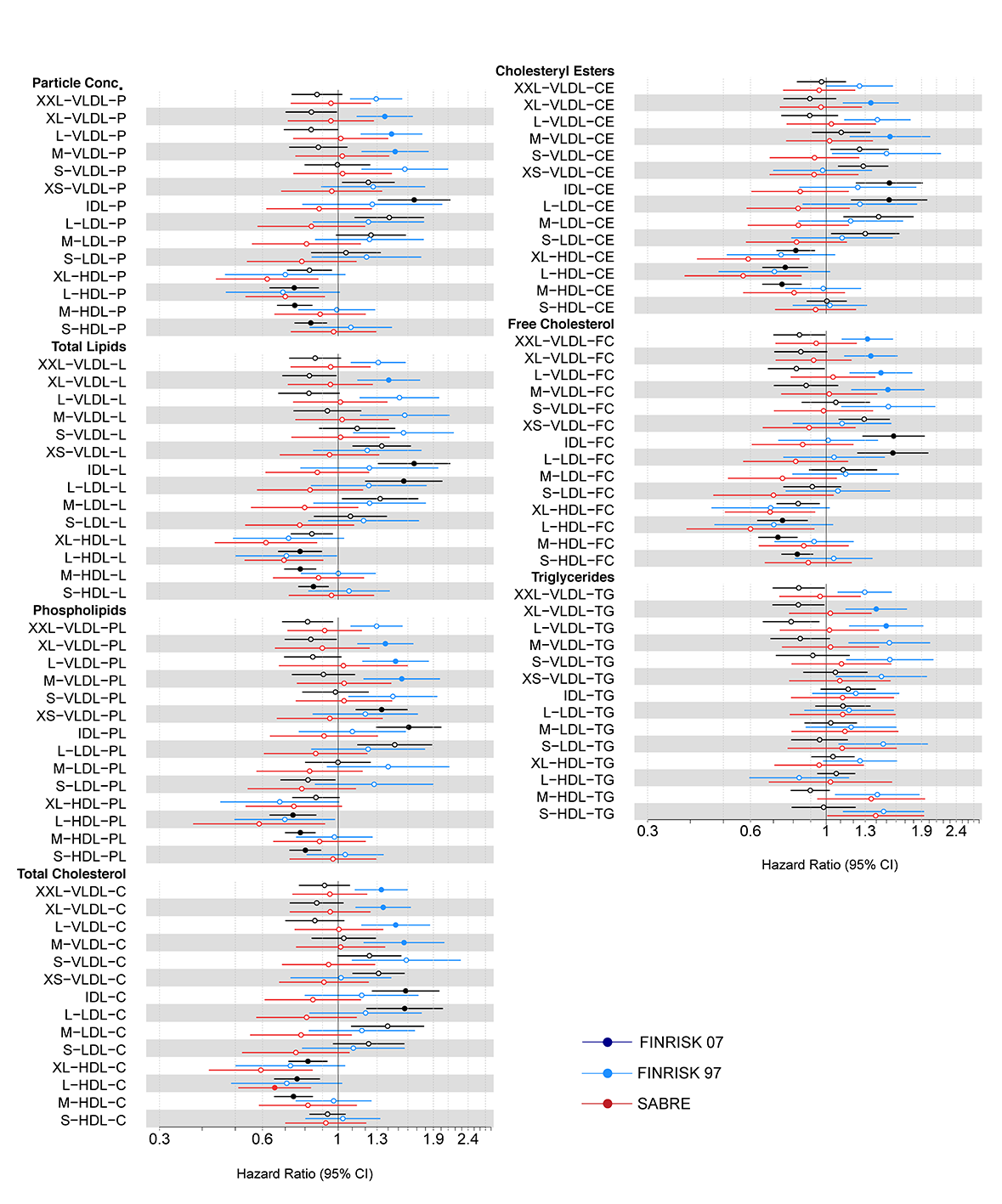

Supplement: S13 Fig — Estimates represent hazard ratios for incident CHD per SD lipoprotein concentration adjusted for traditional risk factors (age, sex, mean arterial pressure, smoking, diabetes mellitus, lipid medication, geographical region in FINRISK, and ethnicity in SABRE), total cholesterol, and total triglycerides. Closed circles represent statistical significance of associations at P < 0.002 and open circles associations that are nonsignificant at this threshold. The horizontal bars refer to the 95% CIs. Underlying data can be found in S2 Data. CHD, coronary heart disease. (TIF) [file pbio.3000572.s017.tif]

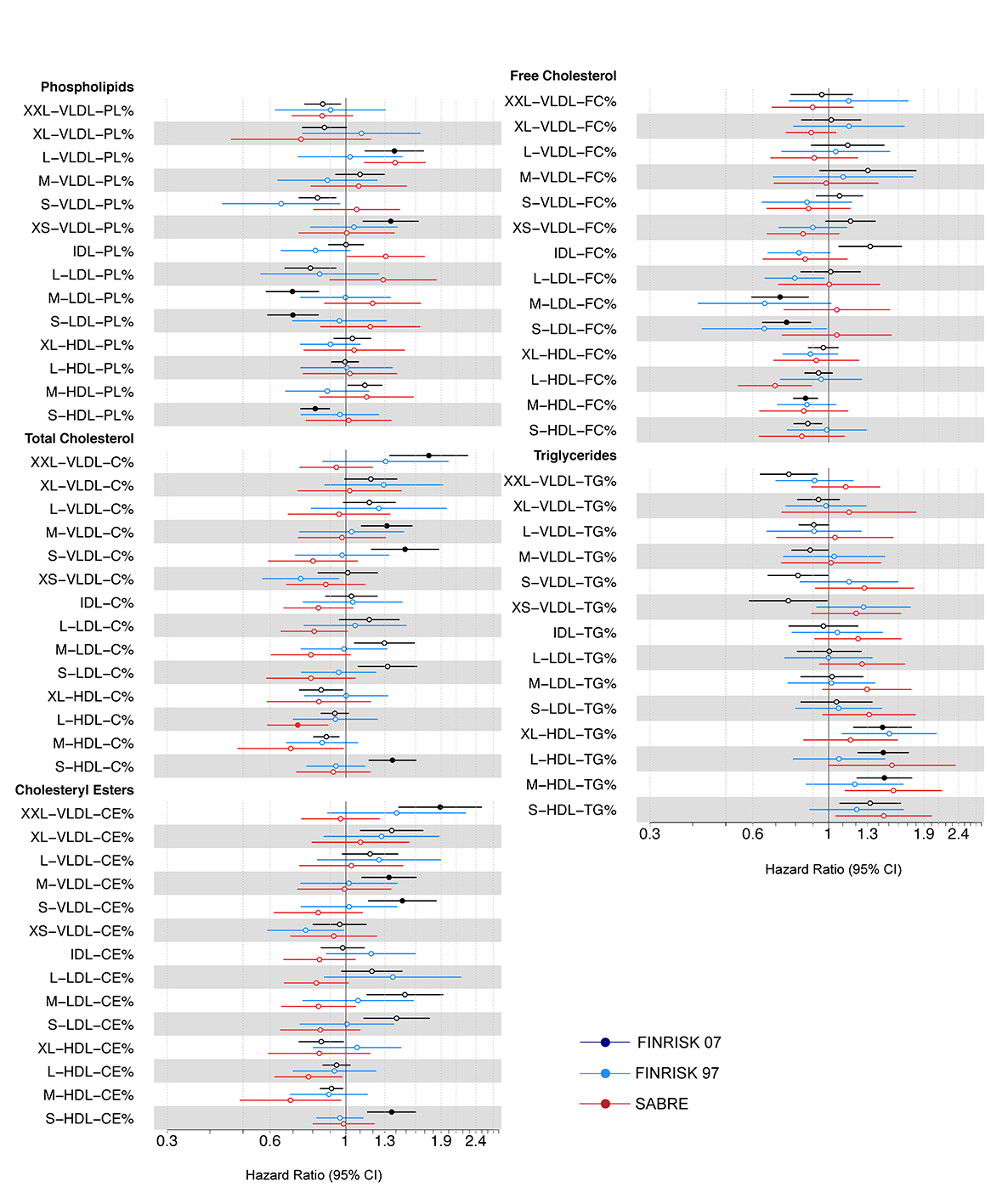

Supplement: S14 Fig — Estimates represent hazard ratios for incident CHD per SD lipoprotein composition measure adjusted for traditional risk factors (age, sex, mean arterial pressure, smoking, diabetes mellitus, lipid medication, geographical region in FINRISK, and ethnicity in SABRE), total cholesterol, and total triglycerides. Closed circles represent statistical significance of associations at P < 0.002 and open circles associations that are nonsignificant at this threshold. The horizontal bars refer to the 95% CIs. Underlying data can be found in S2 Data. CHD, coronary heart disease. (TIF) [file pbio.3000572.s018.tif]

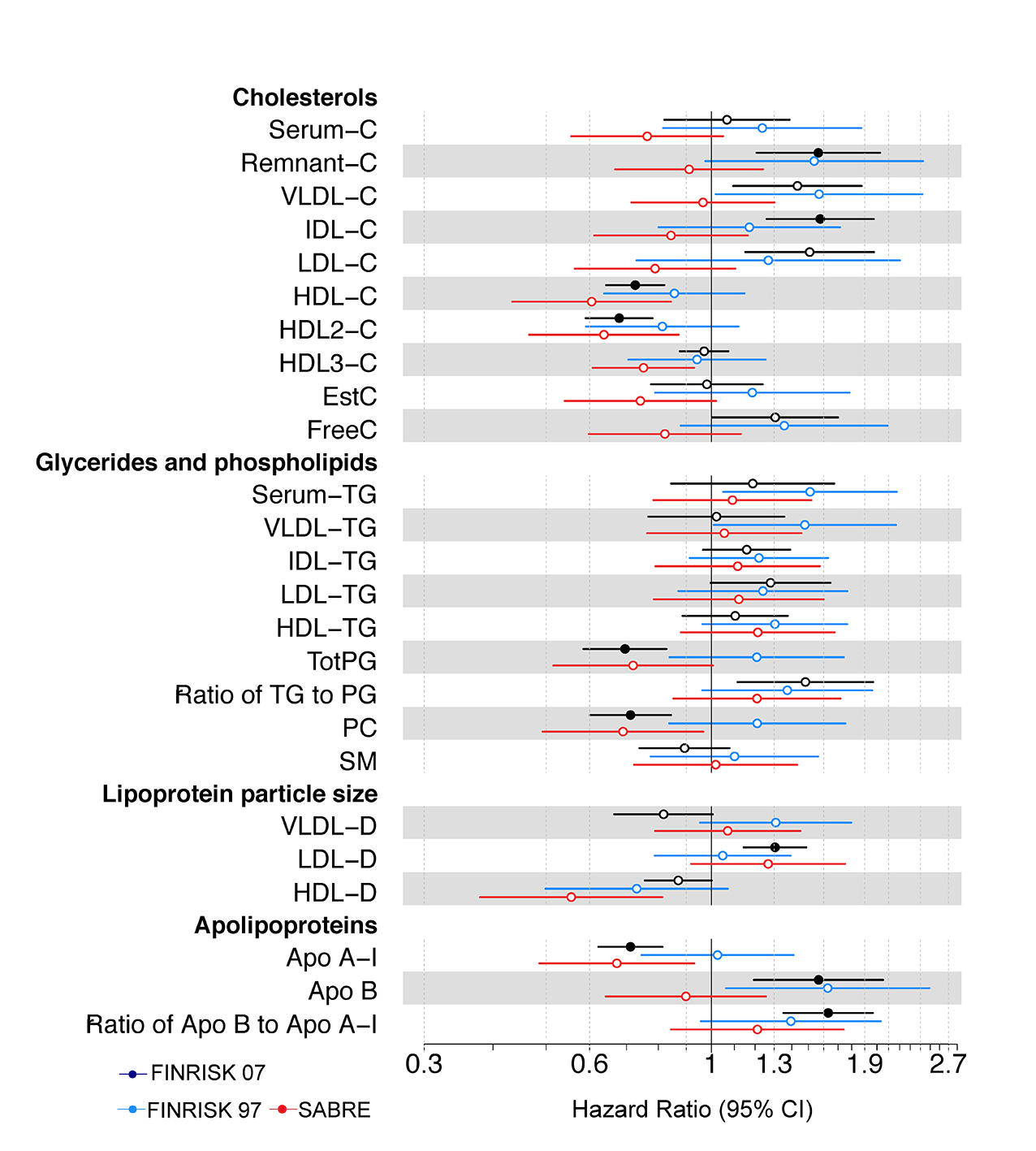

Supplement: S15 Fig — Estimates represent hazard ratios for incident CHD per SD lipoprotein measure adjusted for traditional risk factors (age, sex, mean arterial pressure, smoking, diabetes mellitus, lipid medication, geographical region in FINRISK, and ethnicity in SABRE), total cholesterol, and total triglycerides. Closed circles represent statistical significance of associations at P < 0.002 and open circles associations that are nonsignificant at this threshold. The horizontal bars refer to the 95% CIs. Underlying data can be found in S2 Data. CHD, coronary heart disease. (TIF) [file pbio.3000572.s019.tif]
